# Arsenic detoxification within thermo-alkaline biofilms

**DOI:** 10.3389/fmicb.2026.1783099

**Published:** 2026-04-30

**Authors:** Gwendolyn Cooper, Stephanie H. Ayotte, Martina L. Du, Jessica D. Wood, Breuklyn Opp, Brian Bothner, Brent M. Peyton

**Affiliations:** 1 Thermal Biology Institute, Montana State University, Bozeman, MT, United States; 2 Department of Chemistry and Biochemistry, Montana State University, Bozeman, MT, United States; 3 Center for Biofilm Engineering, Montana State University, Bozeman, MT, United States; 4 Department of Civil Engineering, Montana State University, Bozeman, MT, United States; 5 Department of Chemical and Biological Engineering, Montana State University, Bozeman, MT, United States; 6 Department of Microbiology and Cell Biology, Montana State University, Bozeman, MT, United States

**Keywords:** arsenic, metabolism, metagenomics, microbial mats, Yellowstone National Park

## Abstract

**Introduction:**

The fundamental principles driving community composition and dynamics of microbial mats in thermoalkaline springs are largely uncharacterized. High in not only temperature but also arsenic (As), the microbial populations of Yellowstone National Parks (YNP), USA thermal springs require unique detoxification mechanisms to survive and carry out basic biological functions.

**Methods:**

While many studies have focused on which microorganisms are present, few studies have integrated the use of metagenome sequencing, imaging techniques, and mass spectrometry to gain insight into how structure and function of the mat dwelling organisms might be impacted by the high arsenical species in the ecosystem.

**Results:**

Here, we demonstrate via metagenome sequencing that community composition, including microbial genera *Roseiflexus*, *Thermus*, and *Synechococcus*, and as detoxification abilities change with mat depth and distance from the springs. Arsenical speciation confirmed the generation of bioarsenicals by mat-dwelling microorganisms. Microscopy revealed stratification of microorganisms in the mat, potentially reflecting their arsenic redox capabilities.

**Discussion:**

These data demonstrate how microbial mats are modular, stratified systems that shape and are shaped by environmental and geochemical gradients. Together, these findings characterize novel complexity and associations between geochemical cycles of metals and metabolic adaptations necessary for microorganisms to inhabit thermal springs. In conclusion, these findings demonstrate physiochemical heterogeneity of microbial mats in YNP.

## 1 Introduction

Thermoalkaline springs represent rich, yet understudied environments. With implications for both technological and biological significance, the benefits of studying these unique systems are broad (Felipe Benites et al., 2024). Extreme temperatures in thermal systems are often accompanied by extreme chemical conditions like high or low pH and elevated concentrations of toxic and mobile elements like arsenic (As) (Connon et al., 2008; Wang et al., 2018). Despite harsh environmental conditions, microorganisms can thrive, particularly in the outflow of these hot spring systems. The microorganisms inhabiting these environments present a distinctive microbial ecology that drives biogeochemical processes such as nutrient and metal cycling (Roy et al., 2020; Kozubal et al., 2012).

Substantial research efforts have been focused on utilizing microorganisms in bioremediation of heavy metal contaminated water and soils (Pande et al., 2022). Microorganisms utilize processes like biosorption, bioaccumulation, biotransformation, and bioleaching to survive in environments with high metal concentrations. By maintaining these abilities, microorganisms can become tolerant of environmental extremes. These same techniques have been utilized in bioremediation processes. For example, *Bacillus sphaericus* has an increased capacity for chromium ion biosorption (Velásquez and Dussan, 2009). Microbial attenuation of As transformation have been reported in *Micrococcus* sp. and *Acinetobacter* sp. as well (Tanmoy et al., 2018; Karn and Pan, 2016). This is of particular importance because As is ubiquitous in the environment and represents a significant risk factor for cancer, cardiovascular disease, and other health problems after exposure to contaminated drinking water or contaminated soil (Chung et al., 2014). Therefore, focusing attention on microorganisms inhabiting environments with high arsenic (As) concentrations may provide ways to utilize these organisms for bioremediation efforts.

Evolutionary biology has facilitated large strides in understanding the origin of life (Damer and Deamer, 2020; Russell, 1996; Ward et al., 1998), investigating the complexity of microbe interactions (Wörmer et al., 2020) and understanding mechanisms for As detoxification (Langner et al., 2001) by studying microorganisms in extreme environments. Arsenic (As) is a well-known metalloid that is found in high abundance in geothermal basins around the globe (Leal-Acosta et al., 2010; Maity et al., 2011). Mobilization of As into geothermal waterways is mediated by the available As in the surrounding rock, geochemical conditions, and by the microorganisms that can transform it (Bundschuh and Maity, 2015). In the geothermal springs of Yellowstone National Park (YNP), As concentrations range from 10 μM to as high as 2 mM (Langner et al., 2001).

Outside of YNP, some bacteria have evolved to utilize the more toxic arsenite [As(III)] as an energy source (Santini et al., 2000) whereas others can use the less toxic, arsenate [As(V)] as an electron acceptor during aerobic respiration (Stolz and Oremland, 1999). Gram-negative bacteria confer As resistance by utilizing efflux pumps to transfer arsenic out of the cell thereby lowering intracellular concentrations (Cervantes et al., 1994). The efflux pump, a two-component ATPase complex consisting of ArsA and ArsB subunits, pumps As(III) out of the cell once it has been reduced by ArsC from As(V) to As(III). Additionally, some species can convert highly toxic As(III) to As(V) or to methylated arsenicals such as dimethylarsinous acid (DMA), monomethylarsonous acid (MMA), or arsenobetaine (Tamaki and Frankenberger, 1992), which are then thought to be utilized in the production of larger macromolecules like arsenosugars and arsenolipids. Previous work has shown that most thermal springs discharge As primarily in the form of As(III) and therefore, As(III) represents the dominant species in the source water (Ball et al., 2001; Ball et al., 1998). When source water becomes more exposed to oxygenated conditions by traveling downstream and/or into larger drainages, the amount of As(V) increases (Stauffer et al., 1980). The oxidation of As(III) is likely microbially mediated, since abiotic As(III) oxidation occurs slowly and previous studies have shown microbial populations were responsible for As(III) oxidation (Langner et al., 2001; Wilkie and Hering, 1998; Landrum et al., 2009). Further, it is likely that As oxidation and reduction follow a gradient in both the water system and microbial mats since spatial distributions of different species and ecotypes in microbial mats have been previously documented (Wörmer et al., 2020).

Oxidation of As(III) in YNP thermal springs has been demonstrated (Langner et al., 2001); however, no studies have characterized the geochemical conditions of the water and microorganisms in the Five Sisters Hot Springs System (FS). FS is located in the largest thermal basin in YNP called White Creek Drainage (WCD) (Peach et al., 2022). Many thermal features in WCD maintain distinct, dynamic environments making them of particular interest for cultivating unique microorganisms and improving our understanding of microbial ecology (Peach et al., 2022). Therefore, the aim of this study was to (1) characterize the geochemical signature of FS that influence both As availability and microbial community composition, (2) determine which arsenical species are present in the mat and surrounding water, and (3) spatially characterize the As related genes present in the microbial mat.

## 2 Materials and methods

### 2.1 Site description

**Figure 1:**
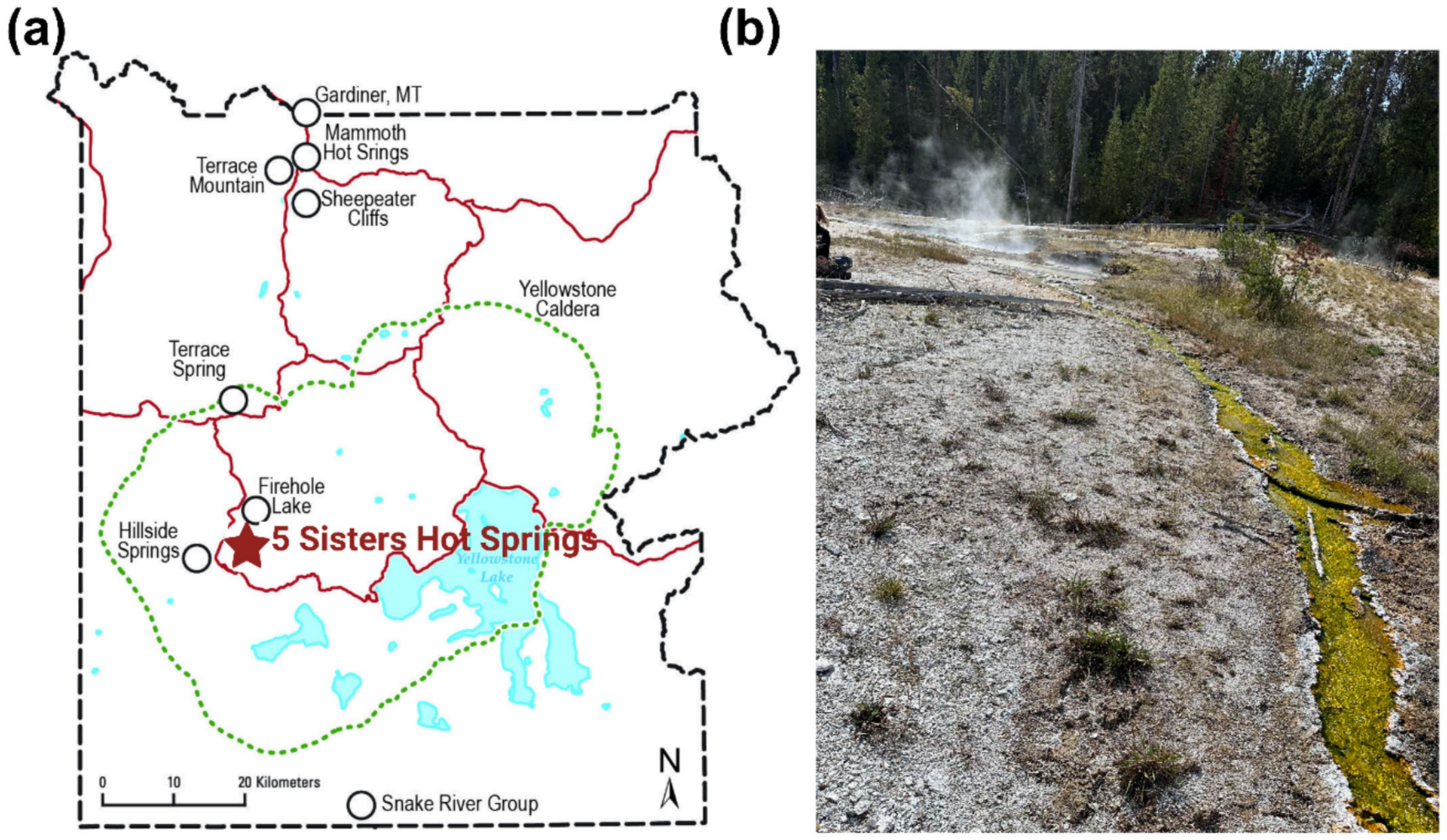
Site Map. **(a)** Map of YNP indicating the location of the 5 Sisters Hot Springs in the White Creek Drainage. Adapted from USGS. **(b)** Picture of the 5 Sisters springs with the outflow channel that was utilized for microbial mat sampling.

The FS site is located on the southeast corner of WCD in YNP (44.532603°N, 110.797282°W) (Figure 1a). The five distinct pools (FS1–FS5) and three distinct geysers of the FS site are found just a few meters from White Creek against a steep hill with a distinct outflow channel (Figure 1b). FS1 is the largest and deepest pool followed by FS5, FS3, FS2, and FS4. FS1, FS2, and FS3 are connected above ground whereas FS4 and FS5 have no above ground connection to the other three pools. Below ground connectivity of the pools remains unknown. FS4 and FS5 are also connected above ground and the FS5 outflow feeds into White Creek. For this study, the outflow of FS5 was studied due to the presence of microbial mats (Figure 1b). Although some microbial mats could be found along the rim of the FS5 pool, these mats were less developed (<1–2 mm in depth) and therefore could not be properly sampled. Five sample sites were identified along the approximately 20 m outflow path that connects the FS pools to White Creek. The initial site (S0) was collected from FS5, the remaining four sites (S1, S2, S3, and S4) were collected at approximately 5 m increments along the outflow.

### 2.2 Aqueous geochemical sampling and analysis

Water samples were collected in September 2023 along the outflow channel of Five Sister’s Hot Spring at five locations and were indicated as Sites (S) 0–4. S0 was in the final hot spring pool of Five Sister’s, otherwise referred to as FS5. Temperature and pH measurements were collected onsite using a combined pH-temperature probe (Hach HQ30d, Hach Co., Loveland, CO, USA). The pH probe was field calibrated using a 3-point buffer calibration in solutions of predetermined concentrations (Fisherbrand™, Fisher Scientific, Inc., Waltham, MA, USA). Calibration verification was conducted periodically using buffer standards. Recalibration was performed if measured values deviated by >0.05 pH units from certified buffer values. Temperature compensation of the probe was enabled during all measurements.

Dissolved oxygen (DO) was determined in the field using HACH method 8,316 dissolved oxygen AccuVac® ampules (HACH® Co., Loveland, CO, USA) and samples were measured with a DR 2700 HACH spectrophotometer (HACH® Co., Loveland, CO, USA). Prior to initial DO sample measurements, the spectrophotometer was zeroed with a field, deionized water blank. Between subsequent measures, a blank check determined any potential drift in the spectrophotometer; if drift was noted, the instrument was recalibrated. DO measurements were performed in duplicate and accepted when replicate values agreed within 5% relative percent difference (RPD); otherwise, samples were reanalyzed. Field duplicates were collected at each site in separate sterile bottles to assess sampling variability. Laboratory analyses were conducted with repeated measures to assess analytical precision. Samples for anion analysis were filtered immediately in the field into sterile 50 mL falcon tubes using pre-rinsed 0.2 μm nylon syringe filters. Samples collected for soluble nitrogen and carbon analysis were filtered immediately using pre-rinsed 0.2 μm regenerated cellulose acetate syringe filters into 24 mL combusted glass vials and filled to ensure there was no headspace. Samples were transported in insulated coolers on ice and maintained at < 4 °C during transport (~2 h) to laboratories at Montana State University (MSU) and then refrigerated at 4 °C until analysis could be completed. All anion analyses were conducted within 48-h of sampling.

Anions were measured on a Metrohm Eco ion chromatograph with a Metrosep A Supp 5150/4.0 column, Metrohm Suppressor Module and 3.20/1.00 mM sodium bicarbonate/sodium carbonate eluent at 0.7 mL/min (Metrohm USA, Riverview, FL, USA). Calibration curves were generated using multipoint standards (R^2^ > 0.995). Laboratory blanks and continuing calibration verification standards were analyzed every 10 samples. The instrument detection limit for all anions was 0.1 mg L^−1^. Analytical precision, assessed via laboratory duplicates, was typically 5 to 10% RPD.

Total carbon (TC; method ASTM D8083), inorganic carbon (IC; method: ASTM D7573), non-purgeable organic carbon (NPOC; method: ASTM D7573), and total nitrogen (TN; method ASTM D8083) were analyzed by MSU’s Environmental Analytical Lab using a Shimadzu TOC-VSH (Shimadzu Scientific Instruments, Inc., Columbia, MD, USA), following ASTM methods. The instrument detection limits for TC, IC, NPOC and TN were 1.5 mg L^−1^, 1.5 mg L^−1^, 0.05 mg L^−1^, and 0.02 mg L^−1^, respectively. Calibration standards curves, method blanks and QC calibration checks were analyzed with each method to verify accuracy and instrument performance. Analytical precision for TN and carbon analyses was between 6 to 10% for all methods.

### 2.3 Microbial mat sampling

**Figure 2:**
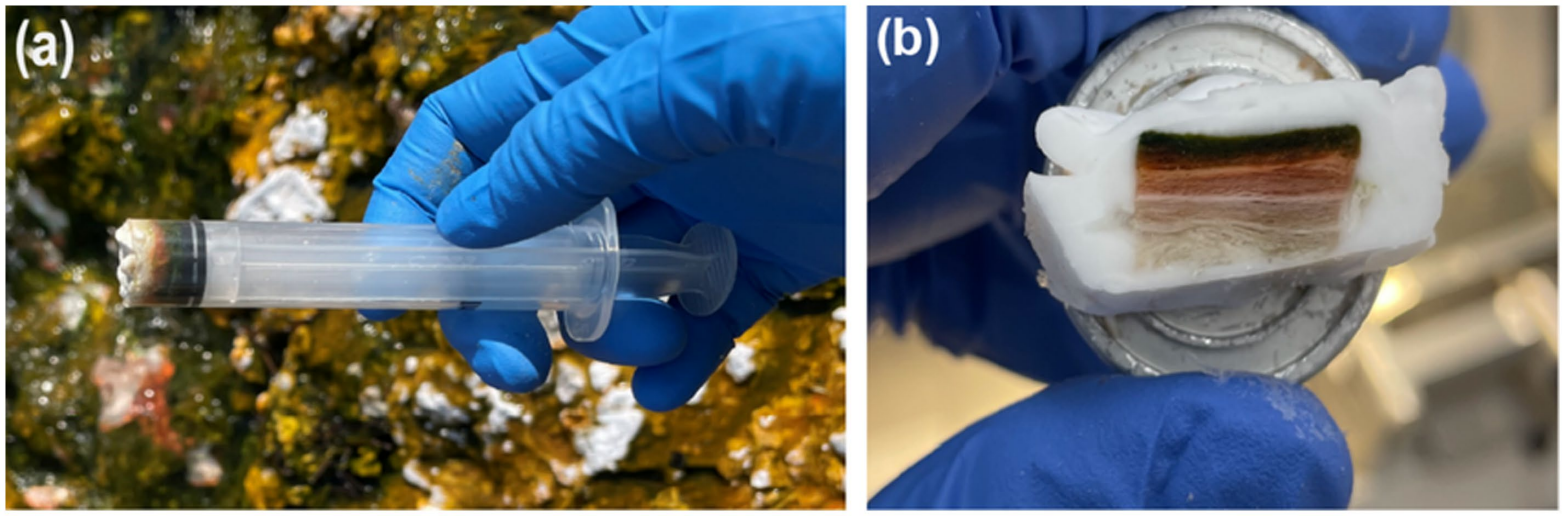
Microbial mat sampling, sectioning, and initial imaging. **(a)** Sampling of microbial mat biofilm for microscopy using a modified 10 mL BD Luer-Lok syringe. **(b)** Microbial mat embedded in optimal cutting temperature (OCT) for cryosectioning.

To preserve microbial mat structure, 10 mL luer lock syringes were modified by removing the luer lock end and sharpening the edge, allowing it to serve as a coring device for collecting mat core samples (Figure 2a). This method enabled the collection of intact mat specimens (including surface biofilm and underlying sediment) while minimizing disruption to native layering. Five mat cores were randomly sampled from S1, S2, S3, and S4. Due to the extremely high temperature (78.7 °C) in S0, no mats were present and therefore could not be sampled. Mat cores were immediately transferred to 15 mL falcon tubes for storage on dry ice in the field. Samples were stored at −80 °C until subsequent analysis.

### 2.4 Staining and imaging

In the field, mat cores were transferred to sterile petri dishes and immediately treated in the field with LIVE/DEAD stain for two hours. This stain is designed to differentiate between live and dead cells based on membrane integrity; however, it failed to produce meaningful fluorescence in these mat specimens. Potential reasons include limited stain penetration due to short incubation time during field sampling. Channels corresponding to LIVE/DEAD fluorescence emission were therefore excluded from subsequent analysis.

Following staining and fixation, the dishes were wrapped with parafilm and transported on ice to MSU. Upon arrival, samples were embedded in optimal cutting temperature (OCT) fluid and frozen using dry ice. The frozen cores were halved using a sterile razor blade, revealing visibly stratified layers and void structures within the mat (Figure 2b). Scotch Permanent Double-Sided Tape was used to stabilize the specimen, prevent splitting at void boundaries, and to anchor it to microscope slides during cryosectioning, thus reducing sectioning artifacts while preserving the flaky and porous nature of the sample.

Brightfield images of the microbial mat sections were taken using a Leica DMi8 THUNDER Imager to obtain a global view of mat structure and ensure sample quality was maintained prior to additional imagining. The same instrument was used to capture fluorescence images to characterize naturally occurring autofluorescence and photosynthetic pigments. Imaging was performed using the microscope’s standard epifluorescence filter channels: Green Fluorescent Protein (GFP), Yellow Fluorescent Protein (YFP), Red Fluorescent Protein (RFP), and Cyanine 5 (Cy5). The microscope’s filter-set designations were used as spectral detection windows for natural autofluorescence and photosynthetic pigments rather than to indicate the presence or expression of fluorescent proteins. No genetically encoded fluorescent reporters were used. Image acquisition and THUNDER Computational Clearing were performed using LAS X software to enhance contrast. All prepared slides and samples were stored at −20 °C prior to imaging.

### 2.5 Arsenic speciation and quantification

Water samples were filtered in the field using an Acrodisc 32 mm syringe filter with 0.8/0.2 μm Supor membrane (Pall Corporation). Arsenic and other metals were liberated from biologic molecules using a 5% nitric acid digestion (Larson et al., 2023). Mat samples were weighed and digested in 70% Optima grade nitric acid (Fischer Scientific) at 65 °C. Digested samples were then diluted in Optima grade water to achieve a final nitric acid concentration of 5%. Once diluted, samples were filtered using an Acrodisc 32 mm syringe filter with 0.8/0.2 μm Supor membrane.

Arsenical species were separated and eluted via HPLC (Agilent SPS 4) using a Hamilton prpx100 column (10 μM pore diameter, 250 mm x 4.1 mm) isocratically with a mobile phase consisting of 10 mM ammonium phosphate and detected via ICP-MS (Agilent 7,800) (Nguyen et al., 2018; Moreira et al., 2011). Each of the arsenic species was quantified using Agilent MassHunter 4.6 (version C.01.06) according to individual arsenic species standard curves prepared in the range of 0.075–1.2 ppm. For arsenic speciation analysis, values were normalized to the volume of sample (cm^3^) digested, after subtraction of digestion blank values. Digestion blanks and blanks containing optima grade water and nitric acid were run every three samples for quality assurance and control. Three biological replicates for both water and mat samples were analyzed. Measurements for the mat and water samples are reported in μg/L. To assess differences between sampling sites, group means for each sample site (S0, S1, S2, S3, and S4) were utilized for subsequent descriptive statistics.

### 2.6 Metagenomic analysis

#### 2.6.1 DNA extraction and shotgun metagenomic sequencing

Microbial mats were aseptically dissected on sterile petri dishes using scalpels disinfected with 70% ethanol (Supplementary Figure S1). Mats were sectioned by dominant color gradations (green, pink and white) and bisected into two separate replicates that were subsequently extracted for DNA. Total community DNA was extracted from samples using the FastDNA™ SPIN Kit for Soil (MP Biomedicals, LLC., Santa Ana, CA, USA) (Saidi-Mehrabad et al., 2020; Ascher et al., 2009). Frozen samples were partially thawed, and approximately 0.5 g of mat material was aseptically transferred to the Lysing Matrix E tubes. The rest of the extraction followed kit manufacturer’s protocol. Extracted DNA was quantified using the Qubit™ dsDNA Broad Range Assay kit (Invitrogen ™ ThermoFisher Scientific, Inc., Waltham, MA, USA). Samples with sufficient, high-quality DNA, with concentrations of 10 ng/μL or greater of eluted DNA, were sent to the SeqCenter, LLC (Pittsburg, PA) for shotgun metagenomic sequencing (1.2 GB per sample).

Library preparation used the Illumina DNA Prep kit with tagmentation and PCR amplification, incorporating custom 10 bp integrated DNA technology dual indices and a target insert size of 280 bp. No size selection or further fragmentation was performed. Sequencing was done on an Illumina NovaSeq X Plus, producing 2 × 151 bp paired-end reads. Reads were demultiplexed, quality checked and adapter-trimmed using bcl-convert v4.2.4 and FASTQC v0.12.1 (Andrews, 2010). Sequence quality did not improve with additional trimming; thus, further sequence trimming was not performed.

#### 2.6.2 Bioinformatic processing and assembly

Reads were first mapped against the human genome reference HG38 (GCF_000001405.40) using Minimap2 v2.26 (Li, 2018) to remove potential human DNA introduced during the sampling, handling and library preparation processes as an initial quality control measure. Low levels of the samples were aligned to the database and discarded from the analysis. Read pairs that did not align to the human reference database were retained for downstream analysis. Taxonomic classification of unmapped reads was conducted using MetaPhlAn (v4.1) with the CHOCOPhlAn Species-level Genome Bins (SGB) database (vJune23). Genus level taxonomic classifications were used for subsequent alpha and beta diversity analysis of communities.

*De novo* assembly of the retained reads was performed using MegaHit v1.2.9 (Li et al., 2016) with default parameters. This step allowed for the reconstruction of contigs from environmental DNA fragments. Quality of the assembled contigs was evaluated with Quast v5.2.0 (Gurevich et al., 2013).

#### 2.6.3 Sample level annotation

Prodigal v2.6.3 (Hyatt et al., 2010) was used to predict Open Reading Frames (ORFs) from assembled contigs, representing putative protein-coding sequences. Predicted ORFs were functionally annotated using GhostKOALA (Kanehisa et al., 2016), which assigns KEGG Orthology (KO) numbers by mapping sequences to the KEGG GENES nonredundant database. KO assignments are determined based on the summation of normalized alignment scores across multiple hits in target genome, allowing classification into orthologous functional groups (Kanehisa et al., 2016). The annotated genes were grouped across samples to create a gene count matrix, with rows representing individual genes and columns corresponding to samples. Each matrix cell reflected the total number of reads assigned to a specific gene within a given sample. Raw gene counts were normalized using DESeq2 v1.40.2 (Love et al., 2014) which applies a median-of-ratios method. For each gene, the count in a sample was divided by its geometric mean across all samples, and the sample-specific median of these ratios was used as a size factor. Normalized counts were derived by dividing each raw count by the corresponding size factor, correcting for sequencing depth and library composition differences.

#### 2.6.4 Metagenome-assembled genome (MAG) analysis

Contigs were grouped into bins representing metagenome-assembled genomes (MAGs) using MetaBAT2 v2.15 with default parameters. Bin quality was assessed with CheckM v1.2.0 (Parks et al., 2015), evaluating the completeness and contamination levels. Based on the minimum required reporting standards, MAGs with over 90% completeness and less than 5% contamination are considered high-quality, MAGs with over 50% completeness and less than 10% contamination are considered medium-quality, and completeness and contamination outside of these ranges are considered low-quality. Of the 212 MAGs recovered, 60 were categorized as high-quality, 52 as medium-quality, and 100 as low-quality according to established minimum genome reporting standards (Supplementary file). MAGs with high and medium quality were further assessed via taxonomic classification and functional potential.

Taxonomic classification of the MAGs was performed using Kraken2 v2.1.3 default Standard Database (RefSeq: archaea, bacteria, viral, plasmid, and human; UniVec_Core). Contig counts and relative abundances were reported to the species level. To ensure reliable taxonomic resolution, only MAGs with ≥70% species-level identity will be discussed taxonomically.

112 MAGs were functionally annotated using the rapid and standardized method established by Bakta v1.8.1 with default parameters and the fifth database version. Bakta leverages Prodigal v2.6.3 to predict coding sequences within a genome. These annotations were used to identify arsenic-related genes and potential arsenic cycling pathways based on KEGG references. Partial pathways were included in the subsequent presence-absence analysis. The Supplementary file includes a complete list of all MAGs sorted by their sample and bin number.

#### 2.6.5 Statistical analysis of metagenomic data

All statistical analyses and visualizations of microbial community structure and gene abundance were performed using R v4.4.2 (R Core Team, 2024). Phylogenetic and ecological metrics were calculated with the vegan package (Oksanen et al., 2022). Merged MetaPhlAn4 relative abundance data was utilized for all taxonomic analysis and because MetaPhlAn4 reports marker-gene-normalized relative abundances, rather than raw counts, analyses were conducted on proportional data and rarefaction was not performed. Taxa were filtered to retain genus-level assignments only. Within-sample relative abundances were normalized to sum to 1. Abundances were aggregated and grouped for genera with a mean relative abundance <1% across all samples into an “Other” category for visualization. Stacked barplots of percent relative abundance were generated using base R graphics.

Technical sequencing replicates were averaged prior to alpha and beta diversity analysis to avoid pseudo-replication. Due to limited biological replication, statistical comparisons are interpreted as exploratory and descriptive rather than inferential. Alpha diversity was quantified using Hill numbers via the hill_taxa() function, and corresponded to richness (q = 0), the exponent of Shannon diversity (Hill-Shannon; q = 1), and the multiplicative inverse of Simpson diversity (q = 2; expressed as effective numbers of taxa) (Jost, 2006; Roswell et al., 2021). A paired Wilcoxon test was used to assess statistical differences across Site or Layer.

Beta diversity (between-sample variation) was explored using Principal Coordinate Analysis (PCoA) based on the Bray–Curtis dissimilarity metric (Kers and Saccenti, 2021). Relative abundance data were Hellinger-transformed prior to distance calculations to reduce influence of highly abundant taxa and account for compositional structure (Legendre and Gallagher, 2001). Bray–Curtis dissimilarity matrices were computed using vegdist() in the vegan package, and PCoA was performed using classical multidimensional scaling (cmdscale()). The percent variance explained by each axis was calculated from positive eigenvalues. Ordinations were generated to visualize sample clustering according to site and layer.

Differences in community composition were evaluated using permutational analysis of variance (PERMANOVA; adonis2(), 999 permutations), with homogeneity of multivariate dispersion evaluated via betadisper(), and permutation testing (permutest()) for Site or Layer (Anderson, 2006). Due to limited biological replication, the number of unique permutations was constrained. Complete enumeration was performed when fewer than 999 permutations were possible, typically 119 permutations. No additional restrictions were applied. Random seeds were fixed for permutation-based analyses to ensure reproducibility, and statistical significance was evaluated at alpha = 0.05.

For functional gene analysis, open reading frames (ORFs) annotated to arsenic cycling pathways via KEGG were subset and normalized as described above. Samples were grouped by site and layer, and the median normalized gene count per group was calculated. These values were log-transformed for visualization. Community-level results were plotted using ggplot2, focusing on trends in arsenic cycling gene presence across spatial gradients. A qualitative MAG-centric analysis was used as a comparative metric for community-based assemblies and their functional potential.

## 3 Results

### 3.1 Aqueous geochemistry

Five sites along the Five Sisters Hot Spring outflow channel, S0, S1, S2, S3, and S4, were measured for pH, temperature, dissolved oxygen, anions and total carbon in duplicate (Table 1). Arsenate [As(V)] and arsenite [As(III)] were measured in triplicate from each location (Table 1). Temperature from the hot springs source water averaged 78.7 °C and decreased along the flow path to approximately 54 °C. Dissolved oxygen (DO) increased along the flow path as well, with initial concentrations of 0.85 ± 0.07 mg O_2_/L from the hot spring source water and increased to 3.07 ± 0.23 mg O_2_/L by S4. The pH maintained moderate alkaline conditions, ranging from 8.56 to 8.74 across all sites. Five Sister Hot Spring, among other springs from the White Creek area in YNP are generally characterized as water dominated, alkaline-chloride thermal systems (Fournier, 1989). Low concentrations of both total nitrogen and non-purgeable organic carbon (NPOC) were observed, and the hot spring was found to have the majority of carbon present in the form of dissolved inorganic species (Table 1).

**Table 1:** Summary of mean (sd) aqueous geochemistry of the five sampled site locations.

Constituent	Location				
	Site 0	Site 1	Site 2	Site 3	Site 4
pH	8.59	8.56	8.54	8.68	8.74
Temperature (°C)	78.7	67.3	64.1	59.3	55.3
DO (mg L^−1^)	0.85 (0.1)	0.97 (0.2)	1.15 (0.1)	2.07 (0.3)	3.07 (0.2)
Cl^−^ (mg L^−1^)	254.9 (0.3)	258.4 (3.5)	256.6 (1.6)	257.3 (1.7)	260.6 (1.1)
F^−^ (mg L^−1^)	22.9 (0.1)	23.0 (0.0)	23.1 (0.0)	23.3 (0.1)	23.6 (0.3)
SO_4_^3−^ (mg L^−1^)	17.0 (0.1)	17.1 (0.0)	17.2 (0.0)	17.4 (0.1)	17.6 (0.2)
NPOC (mg L^−1^)	0.259 (0.01)	0.258 (0.00)	0.461 (0.09)	0.352 (0.04)	0.273 (0.05)
IC (mg L^−1^)	54.9 (0.1)	54.8 (0.0)	55.6 (0.3)	54.6 (0.8)	53.6 (0.3)
TC (mg L^−1^)	55.13 (0.4)	55.23 (0.0)	56.16 (0.0)	56.97 (1.1)	56.41 (0.5)
TN (mg L^−1^)	0.040 (0.00)	0.035 (0.00)	0.057 (0.01)	0.036 (0.01)	0.025 (0.01)
As(V) (μg L^−1^)	392.920 (44.7)	473.095 (4.7)	611.479 (4.1)	582.930 (108.4)	528.569 (40.1)
As(III) (μg L^−1^)	39.522 (1.4)	47.805 (1.8)	43.351 (0.4)	29.375 (4.9)	18.084 (0.8)

### 3.2 Arsenic speciation and quantitation

**Figure 3:**
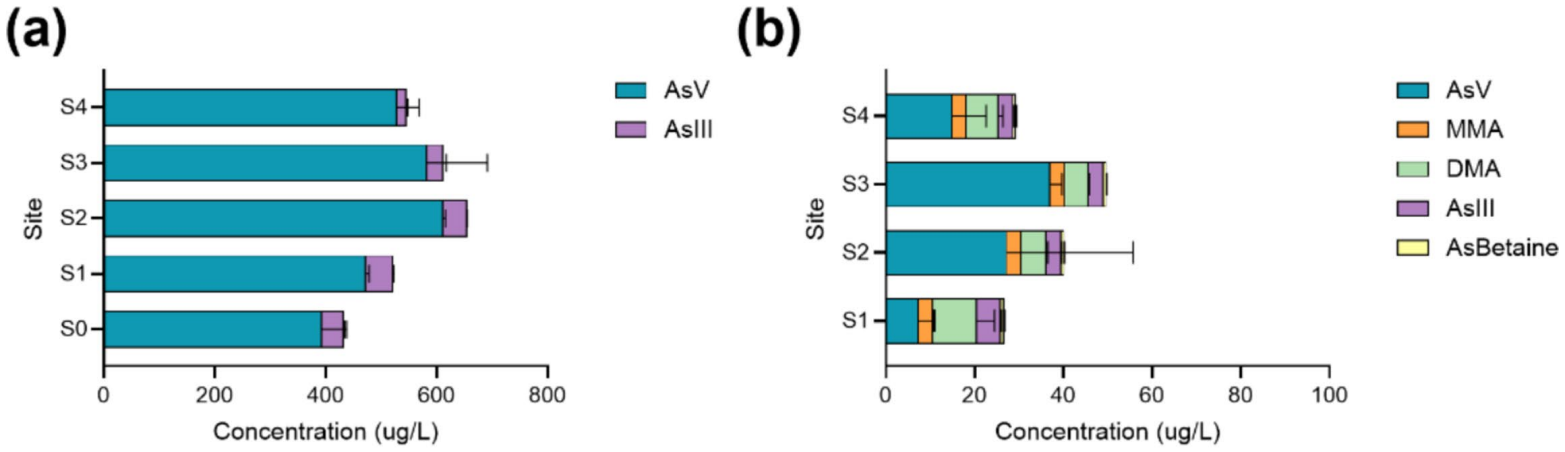
Average arsenical concentrations. Arsenical concentrations of As(V), As(III), dimethylarsinous acid (DMA), monomethylarsonous acid (MMA), and arsenobetaine (AsBetaine) in water **(a)** and in mats **(b)** reported in μg/L from the sites at 5 Sisters Hot Springs.

To determine the concentration of arsenical species in the water and microbial mats at the FS, arsenic speciation was performed via ICP-MS. Arsenic speciation tracked As(V), As(III), dimethylarsinous acid (DMA), monomethylarsonous acid (MMA), and arsenobetaine (AsBetaine). Interestingly, there were no significant differences in the concentrations of arsenicals in the water between any of the sites (Figure 3a) nor were there any correlations between As species and dissolved oxygen. Although dissolved oxygen increased in a linear fashion between the sites (Table 1), this trend was not observed for As(V) or As(III). Additionally, As(V) and As(III) were the only arsenical detected in the water. This finding is in stark contrast to arsenical species and concentrations within the microbial mat samples. Sites S1 and S3 as well as sites S3 and S4 exhibited significant differences (*p* < 0.001) in As(V) concentrations (Figure 3b). Sites S3 and S4 also differed significantly in the levels of MMA and DMA detected (*p* < 0.05) (Figure 3b). The apparent absence of transformed arsenicals (DMA, MMA, and AsBetaine) in the water but not in the mat samples indicated that all transformed arsenicals are produced by mat-dwelling organisms. Furthermore, the differences in concentrations and species of arsenicals within the mats located at the various sampling sites may suggest differential niche partitioning of mat dwelling organisms. To determine if microbial composition differed within the mats themselves as well as between sampling sites, mats were dissected for DNA extraction and shotgun metagenomic sequencing.

### 3.3 Diversity of mat biofilm communities

**Figure 4:**
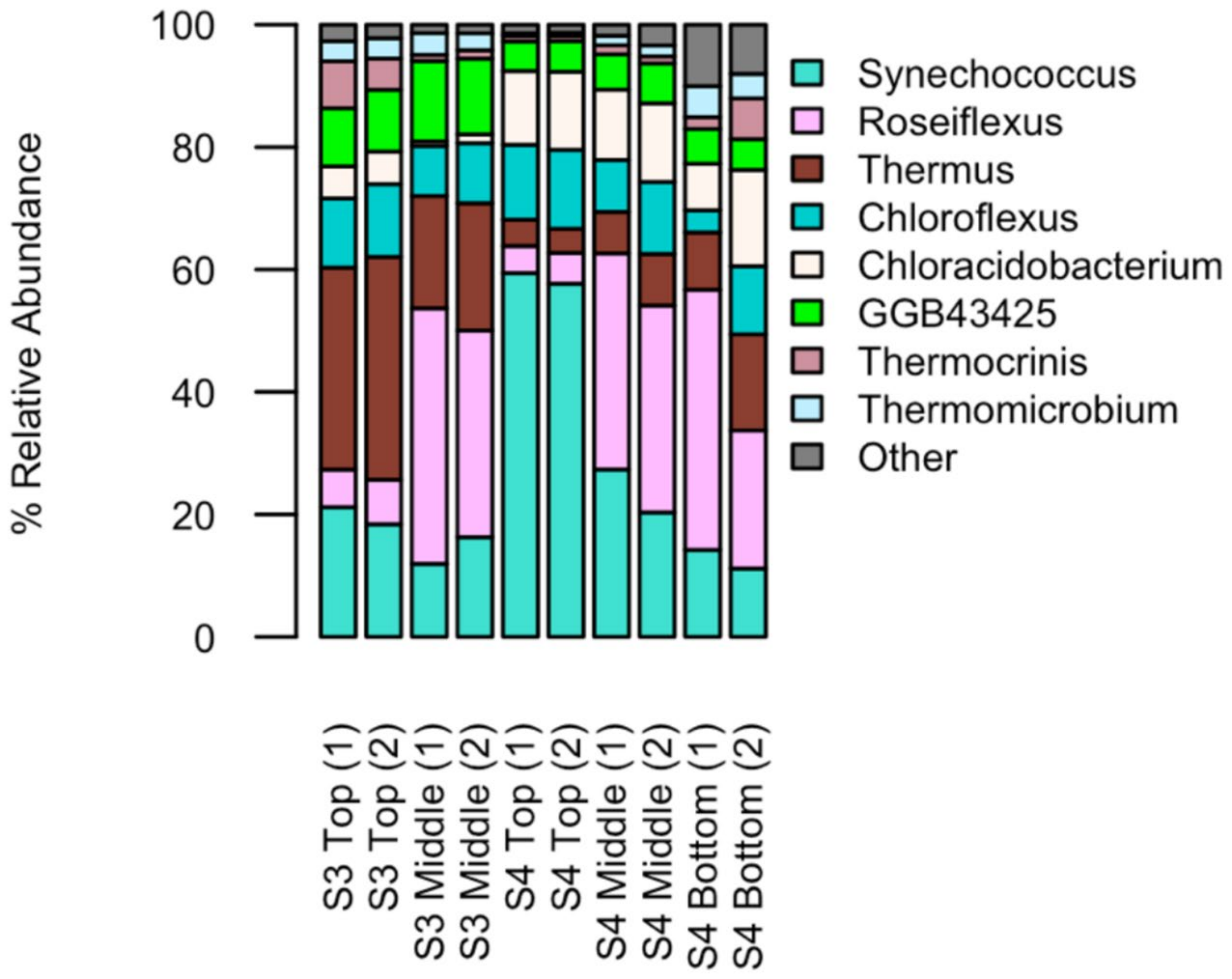
Relative abundance (>1%) from the genera assigned by taxonomic classification of unassembled reads grouped by site and sample layer.

The most prevalent genera (>1%) across biofilm samples were *Synechococcus* (25.8% ± 17.0%)*, Roseiflexus* (23.2% ± 15.2%)*, Thermus* (15.7% ± 10.9%)*, Chloroflexus* (10.1% ± 2.6%)*, Chloracidobacterium* (8.5% ± 4.9%)*, GGB43425* (7.8% ± 3.0%)*, Thermocrinis* (2.8% ± 2.5%), and *Thermomicrobium* (2.6% ± 1.4%), with the remaining 3.3% ± 2.9% attributed to other genera (Figure 4). *Synechococcus* (11.1–59%) and *Roseiflexus* (5.1–42.9%) were generally dominant, together accounting for nearly half of the community in some layers (Figure 4). *Roseiflexus* was more abundant in the middle and bottom layers (22.5–44.5%) than the top layers (4.4–7.2%). MAG analysis confirmed the presence of *Roseiflexus* sp. *RS-1* in the top and middle layers, but abundance was not assessed. *Thermus* was more abundant in S3 (18.3–36.4%) than S4 (3.9–0.7%), while *Chloracidobacterium* showed the opposite trend (S4: 7.6–15.7%; S3: 0.7–5.3%).

**Figure 5:**
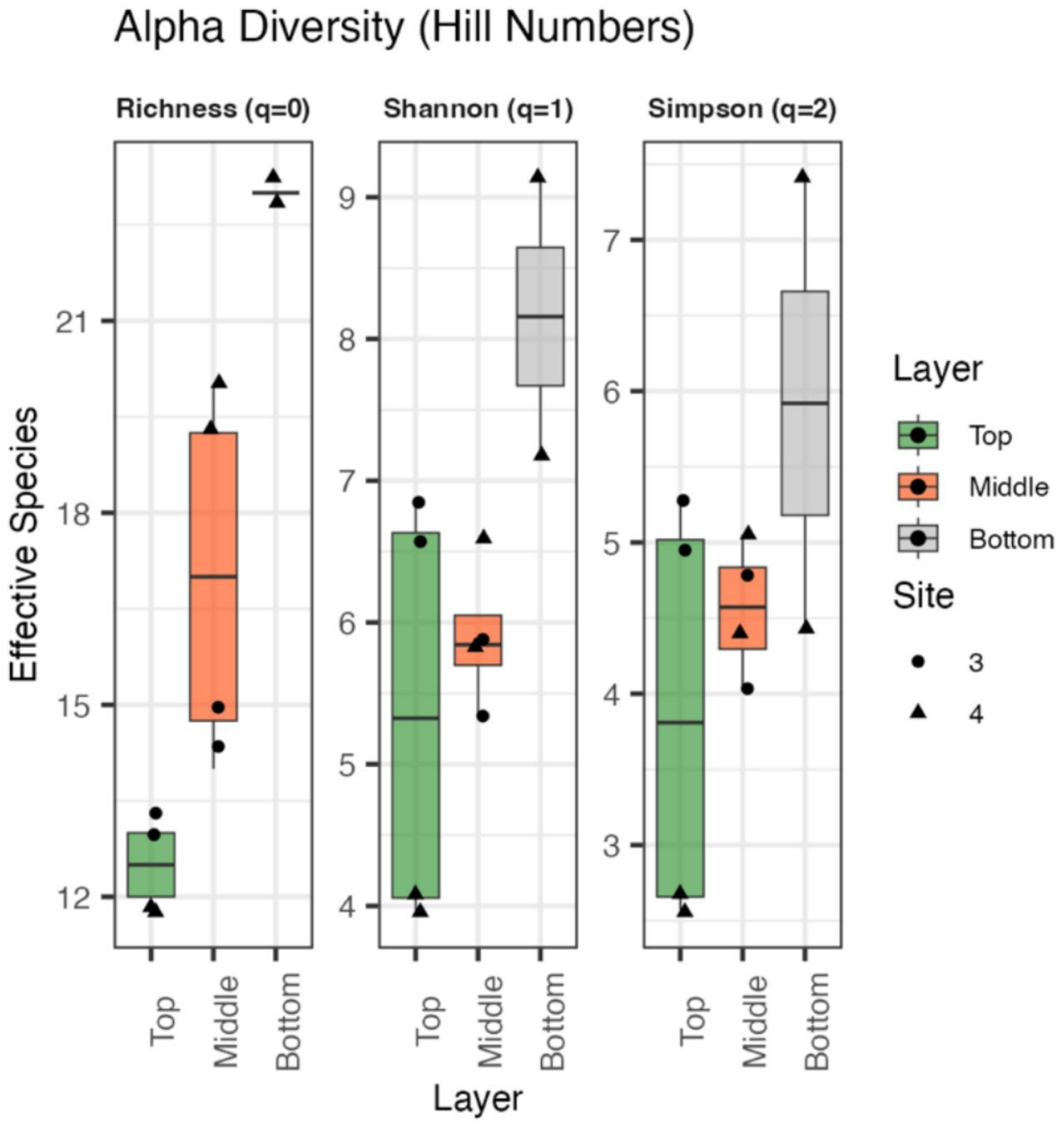
Alpha diversity values calculated using Hill numbers for richness, Hill-Shannon diversity index, and Hill-Simpson index for the top, middle, and bottom layers of the biofilm.

Due to limited mat biomass, only S3 (top and middle) and S4 (top, middle and bottom layers) were sequenced. Alpha diversity metrics were calculated using Hill numbers for richness, Hill-Shannon and Hill-Simpson diversity, (Figure 5). Technical replicates revealed minor variability, particularly in the S4 bottom layer. Richness increased with depth across the biofilm; though differences between layers were not statistically significant (*p* > 0.05). Overall, S3 exhibited higher effective diversity than S4, with the top layer showing the greatest Hill-Shannon and Hill-Simpson values. Differences across layers were smaller in S3 than S4. Results suggest potential vertical structuring of diversity, which warrants further study with increased sampling.

**Figure 6:**
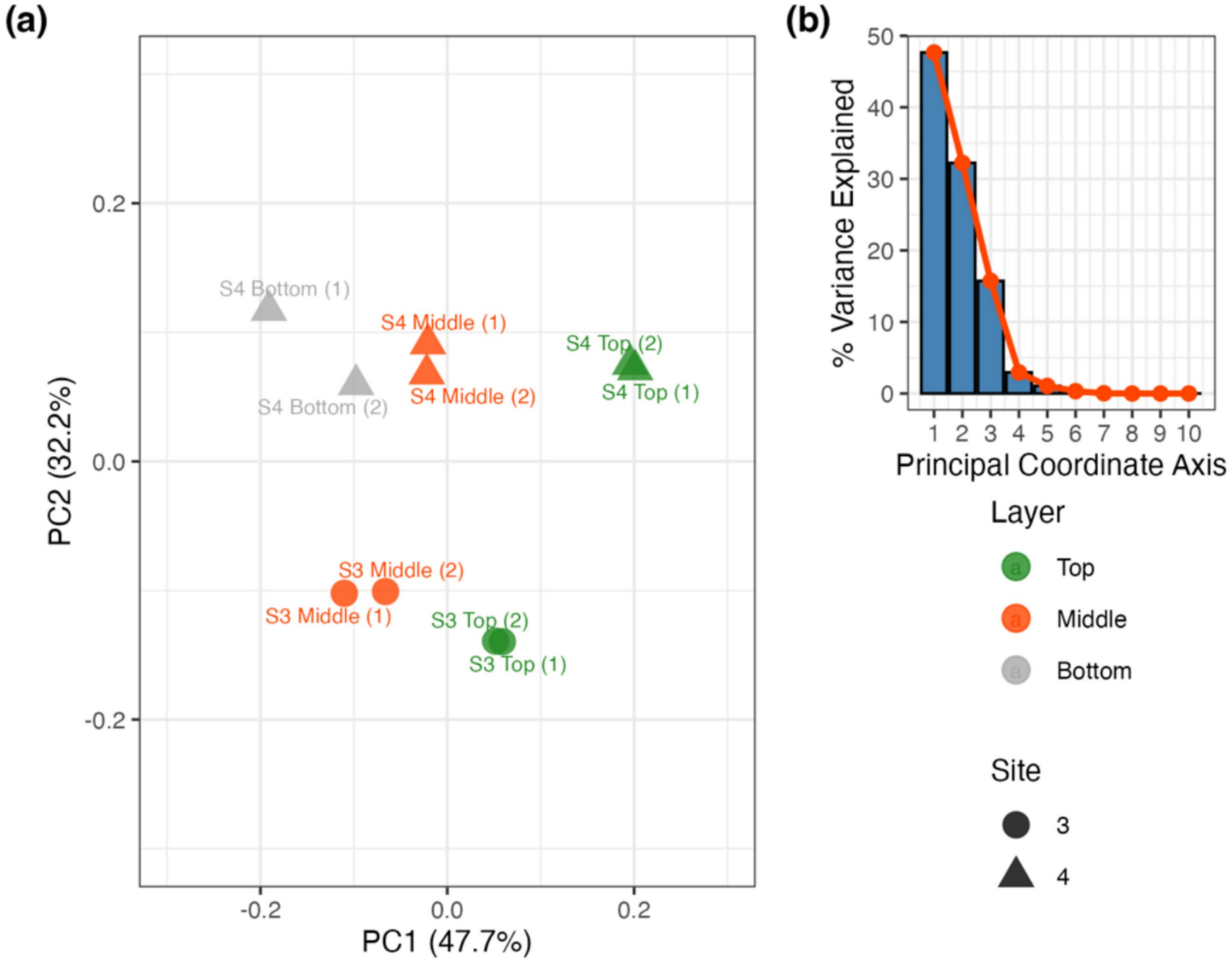
Principal coordinate analysis of the mat sample layers from S3 and S4: **(a)** PCoA of S3 and S4 grouped by layer and site and **(b)** the corresponding scree plot.

PCoA of Hellinger-transformed relative abundances (Bray-Curtis) indicated clustering by Layer along PC1 and Site along PC2, suggesting potential compositional stratification. However, these findings were not statistically significant (Figure 6a; p > 0.05). Significant differences in dispersion were observed only among layers (*p* < 0.05), indicating within-group variation. Given the low sample replication, these ordinations are interpreted as qualitative.

#### 3.3.1 Functional potential of mat biofilms for arsenical-cycling

**Figure 7:**
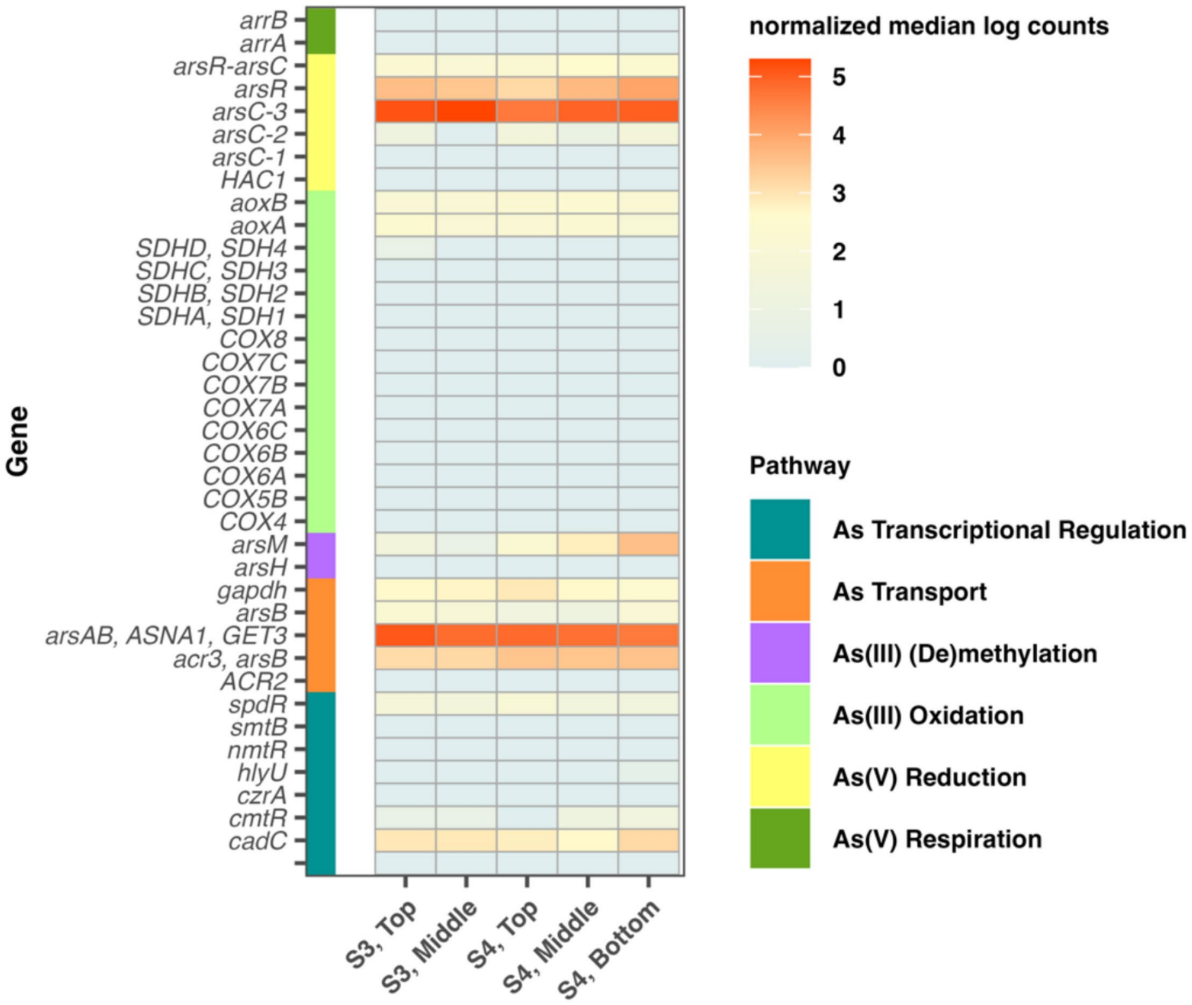
Normalized arsenic gene counts at Sites 3 and 4 from the top, middle, and bottom layers of the biofilm mat. Genes are grouped by arsenic pathways.

Genes associated with the small and large subunits of the As(III) oxidase operon (*aoxA/aoxB*; now *aioA/aioB*) were identified in all sites and layers, with relatively uniform normalized median abundances across all sites (Figure 7).

Two forms of As(V) reductase were identified (*arsC* thioredoxin- and glutaredoxin-dependent). Thioredoxin-dependent *arsC* gene exhibited substantially higher normalized abundance across all sites and layers compared to the other variant, with the S3 top and middle layers elevated relative to other samples (Figure 7).

The arsenite efflux transporter gene, *acr3,* and arsenite-transporting ATPase gene, *arsA,* were detected in all samples. S4 exhibited higher normalized counts of *acr3* compared to S3. In contrast, *arsA* showed greater abundance in the top and middle layers of both S3 and S4 relative to the bottom layer. Additionally, arsenite methyltransferase, *arsM*, was predominantly observed in S4, particularly in the bottom layer.

High-quality MAGs included *Roseiflexus* sp. *RS-1*, *Themomicrobium roseum*, *Thermocrinis ruber*, *Thermus aquaticus*, and *Chloracidobacterium aggregatum* (Supplementary file). Other high-quality MAGs lacked confident species-level assignments and are therefore only discussed in reference to functional potential. Approximately 57% of high- and medium-quality MAGs contained genes associated with arsenic cycling, including oxidation, reduction, transport or (de)methylation pathways. MAGs from S3 (top: 80.9 ± 1.29%; middle: 75.7 ± 6.06%) demonstrated greater overall arsenic cycling potential compared to S4 (top: 52.8 ± 3.93%; middle: 52.3 ± 3.21%; bottom: 43.7 ± 5.21%).

### 3.4 Microscopy of microbial mat

**Figure 8:**
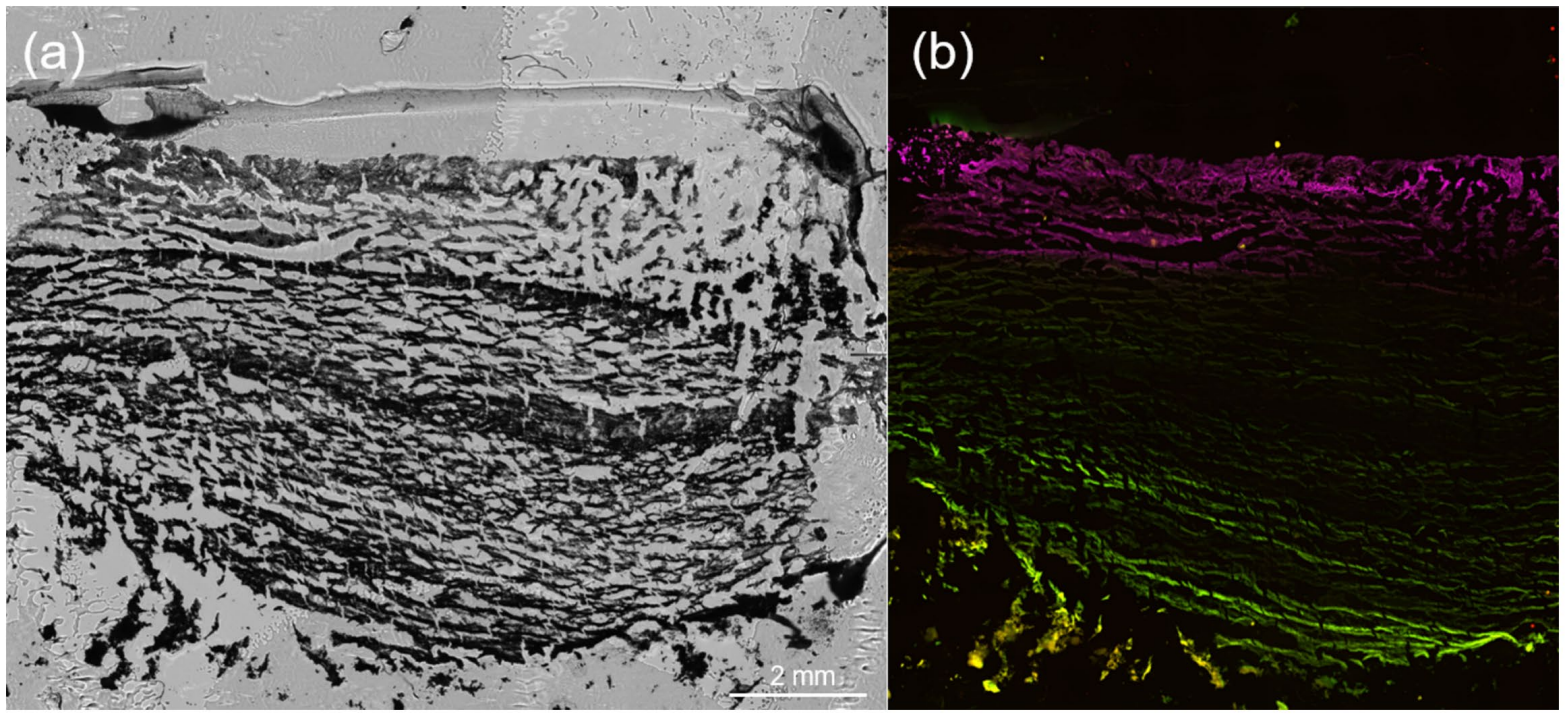
Microscopy images of a cryosectioned microbial mat sample from S4 the Five Sisters Hot Springs. Samples were stained using LIVE/DEAD, though no reliable fluorescence was observed from the stain. **(a)** Brightfield image of the cryosectioned mat. **(b)** Fluorescence signals were captured using Cy5, GFP, RFP, YFP filters, reflecting natural autofluorescence and pigment emissions. Structural layering and spectral separation indicate metabolic zoning and potential microbial interactions within the mat. The magenta layer represents the top of the surface of the mat along the *z*-axis.

Brightfield images of the microbial mat provide insight into structure and highlight the flaky, porous morphology of the mat (Figure 8a). Fluorescence microscopy with Cy5, GFP, RFP, and YFP filters enabled the differentiation of microbial populations within the Five Sisters Hot Springs microbial mats based on their natural fluorescence (Figure 8b). Each filter captured light within a specific emission wavelength range that aligned with the known autofluorescent properties of certain microorganisms.

LIVE/DEAD staining was initially conducted to evaluate microbial viability, but the results were inconclusive. The staining reagents failed to generate distinct fluorescence signals, likely due to the mat’s dense biomass and flaky morphology, which can hinder stain penetration. As a result, fluorescence data from the viability stain were excluded from the final analysis.

## 4 Discussion

The waters from Five Sisters hot spring are characterized by their alkaline-chloride nature, with temperature and pH ranging from 55.3–78.7 °C and 8.54–8.74 in the outflow channel of FS5, respectively (Table 1). High concentrations of chloride (255–261 mg/L), fluoride (23–24 mg/L), and arsenic (450–700 mg/L) are attributed to high-temperature rock-water interactions with rhyolitic bedrock (Colman et al., 2024; Kostešić et al., 2023). Predominate microorganisms in such an extreme environments have been identified based on targeted gene sequencing (Podar et al., 2020) and shotgun metagenomic sequencing methods (Colman et al., 2024). To date, only two studies have investigated FS hot spring using advanced sequencing methods, including 16 s rRNA targeted amplicon sequencing (Peach et al., 2022) and shotgun metagenomics sequencing (Wood et al., 2024) to discern microbial community diversity. To our knowledge this is the first study to apply shotgun metagenomic sequencing to FS outflow biofilms alongside elemental analyses and microscopy to assess the functional potential for arsenical detoxification.

Alpha diversity patterns across sites and layer suggests that microbial communities are structured by physicochemical gradients in the outflow channel. The highest diversity was observed in bottom-layer samples from S4, potentially due to their gravellier media. DNA extraction and sequencing potentially had more varying success compared to the more organic-rich samples from other layers. Additionally, extracellular DNA from dead cells have been observed to play a role in biofilm structure and development, serving as a nucleation site for new growth (Bayles, 2007). This genomic DNA may settle more readily to the lower reaches of the biofilm resulting in higher regional alpha diversity and overall increased variability of samples in the bottom layers.

Elevated diversity was additionally observed in regions with lower temperatures and higher oxygen availability (Figures 4, 5); consistent with known effects of oxygen and redox gradations on microbial metabolism, enzyme activity and nutrient availability (Ospino et al., 2019; Malina et al., 2021; Tian and Hua, 2010). Fluorescence *in situ* hybridization (FISH) and microsensors have confirmed that oxygen and pH gradients play a role in community structural differences (de Beer et al., 1994; Suarez et al., 2019). Oxygen gradients alter redox potentials within biofilms and result in growth of aerobic organisms closer to the surface and the potential to readily oxidize toxic compounds to less harmful by-products (Chen et al., 2017). Further, at these pH values and temperatures below approximately 72 °C, sunlight penetration contributes to biofilm development, with phototrophs colonizing closer to the surface (Perillo et al., 2025). This has readily been observed in many thick biofilms, particularly in YNP, with a green, top layer of the mat composed almost entirely of cyanobacteria (Wood et al., 2024). Across FS5 samples, dominant genera included *Synechococcus, Thermus* and *Roseiflexus* (Figure 4; Supplementary file).

Our findings suggest that site location relative to the source hot spring water may influence community composition more than the vertical layer within the biofilm (Figure 6). Site-dependent differences in nutrient availability, temperature, dissolved carbon and dissolved oxygen (Table 1) likely drive niche partitioning along the FS outlet stream (Peipoch et al., 2019; Malard and Guisan, 2023). These community differences are further reflected in the metagenomic functional potential, indicating spatially structured capacities for arsenical cycling and detoxification within the biofilm.

### 4.1 Functional potential

Arsenic metabolism appears to be broadly integrated into the microbial ecology of the FS outflow biofilms. The widespread and relatively uniform distribution of *aox/aio* genes across sites and layers suggests that arsenite oxidation may serve as both detoxification and potential energy-yielding functions in the system (Andres and Bertin, 2016). The arsenate reduction pathway, involving the *ars* operon which is controlled by the ArsR transcriptional repressor, reduces As(V) to the more toxic and mobile As(III) (Song et al., 2022). Nearly every microbe reportedly has ArsB or Acr3 efflux permeases which actively transport As(III) out of the cytoplasmic membrane for cellular detoxification (Song et al., 2022). Gene families including *arsC, acr2* and *GstB* reduce As(V) in the cytoplasm and then excrete As(III) using the ArsB or Acr3 efflux pumps (Song et al., 2022). Our data indicate that microorganisms within each mat section maintain genes for arsenate reduction, reflective of their collective need to remove intracellular As (Figure 7). The ArsC enzymes are either thioredoxin or glutaredoxin dependent (Messens and Silver, 2006) and even less commonly, NADPH dependent (Ordóñez et al., 2009). Many ArsC enzymes are linked to the thioredoxin (Trx) system as electron donor sources for the catalyzation of arsenate reduction and have been observed to be a more common pathway compared to glutaredoxin- and NADPH- dependent ArsC systems (Ordóñez et al., 2009). Communities observed in the FS outflow channel follow this paradigm, with greater observed counts of thioredoxin-dependent *arsC* (Figure 7, *arsC-3*) compared to glutaredoxin (*arsC-2*) and NADPH dependent (*arsC-1*) genes.

As(III) oxidases, including AioAB and ArxA, are the primary enzymes for As(III) oxidation (Song et al., 2022; Andres and Bertin, 2016; Hurst, 2022; Keren et al., 2022). The expression of *aio* genes is regulated by a two-component signal transduction system consisting of a sensor histidine kinase (AioS) and a transcriptional regulator (AioR) which sense the presence of As(III) in solution (Shi et al., 2020; Hurst, 2022; Zhu et al., 2017). The resulting catabolic oxidation of As(III) supports chemolithoautotrophic growth in the presence of electron acceptors such as oxygen or nitrate (NO_3_^−^) (Shi et al., 2020; Song et al., 2022; Andres and Bertin, 2016; Hurst, 2022; Zhu et al., 2017; Visscher et al., 2020). Increased dissolved oxygen levels in the surrounding outflow of FS indicate the possibility for biologically driven oxidation processes in the mat biofilm (Table 1). Due to changes in oxygen availability within biofilms, As(III) oxidation likely occurs near the surface at the water-biofilm interface and phototrophic layers where oxygen is most prevalent (de Beer et al., 1994). This was confirmed via our MAG analysis which identified the presence of potential arsenic related oxidation capabilities in organisms located in the top and middle layers of S4 only (Supplementary file). S4 additionally corresponded to the highest measured dissolved oxygen concentrations along the outflow of FS (Table 1). Despite samples displaying seemingly consistent normalized abundances of *arxA* and *arxB* genes throughout sites and layers (Figure 7), the identification of genes through MAG analysis may indicate a more nuanced reality in which organisms closer to oxygen rich waters play a more dominant role in arsenite oxidation processes.

The presence of the arsenite methyltransferase, *arsM*, which catalyzes the methylation of As(III) to less toxic arsenicals like MMA and DMA, was observed in predominantly S4, with increased abundance in the bottom layer (Figure 7). Since deeper water is less exposed to oxygen, concentrations of As(III) are likely higher than As(V) (Stauffer et al., 1980; de Beer et al., 1994). Therefore, microorganisms inhabiting the bottom layers of the mat are likely to be exposed to higher levels of As(III). Ultimately, this results in a demand for proteins, such as *arsM*, which detoxify their environment. The high abundance of *arsM* in the deeper sections of the mat suggest that geochemical conditions of As and O_2_ abundance are large drivers of microbial mat community composition. Future analysis should investigate this hypothesis by performing more detailed layer specific As speciation and omic-related analyses.

Respiration of As(V) by prokaryotes utilizes As(V) as a terminal electron acceptor for energy generation (Drewniak and Sklodowska, 2013), and the *arrA* and *arrB* As respiratory gene families reduce As(V) to the more toxic As(III) (Song et al., 2022). The overall absence (lack of detection) of this respiration pathway indicates that the microbial mat at FS does not use As(V) as an electron acceptor for energy and that resistance and detoxification pathways, including (de)methylation, oxidation and reduction are their primary mechanisms for handling toxic arsenic concentrations. The absence of these gene families within the microorganisms of the FS mat system suggests there are multiple pathways microorganisms may be utilizing for As detoxification (Figure 6).

### 4.2 Environmental gradients guide microbial community distribution

To investigate microbial mat composition with minimal disturbance of mat structural integrity, samples were embedded in OCT, cryosectioned, and imaged. The inability to verify cell viability through LIVE/DEAD staining demonstrates the challenges of applying certain stains to structurally complex, pigmented systems. Natural autofluorescence captured via spectral filters offered robust structural data without the need for exogenous dyes.

The observed fluorescence patterns reflect both the chemical properties of microbial pigments and the ecological characteristics of the organisms present (Figure 8). Microscopy images revealed layers within the mat, with distinct autofluorescent populations observed in close spatial proximity (Figure 8; Supplementary Figure S2). Green fluorescence, detected using the GFP and YFP filters, was attributed to filamentous anoxygenic phototrophs such as *Chloroflexus*, which produce bacteriochlorophylls that naturally emit within this range (Malina et al., 2021). Red to far-red signals, observed through the RFP and Cy5 filters, were linked to zones likely dominated by aerobic thermophiles like *Thermus*, which contain carotenoid pigments responsible for red autofluorescence (Tian and Hua, 2010). While autofluorescence alone does not confirm physiology, such spatial arrangements are consistent with the potential for metabolic interactions. Although LIVE/DEAD results were inconclusive due to the hindering of stain penetration, likely as a result of the denseness of the microbial mat and its flaky morphology, autofluorescence imaging using spectral filters remained effective for imaging microbial spatial organization and highlighting differences in community composition within the mat. Overall, these results are in agreement with the metagenomic sequencing which demonstrate community composition varies with depth of the mat (Figures 5, 7). When taken together with the genomic functional potential to oxidize/reduce As (Figure 6) in the mat, it is possible that As could be a contributing factor to the spatial segregation of microorganisms within the mat.

### 4.3 Arsenic oxidation–reduction is microbially mediated

To determine differences in arsenical concentrations in the FS water and microbial mats, arsenic speciation was conducted via ICP-MS. As oxidation and reduction can occur both abiotically and biotically. Inorganically, As(III) is oxidized to As(V) in the presence of an electron acceptor – commonly via oxygen, manganese oxides, or certain iron compounds (Daoyan et al., 2022). Conversely, As(V) is abiotically reduced to As(III) in the presence of ferrous iron, or via hydrogen sulfide (H_2_S) in anoxic environments. Biotically, As reduction and oxidation reactions can be carried out by numerous microorganisms which may directly or indirectly impact the As biogeochemical cycle by altering As mobility, toxicity, and distribution (Yin et al., 2022). Realistically, an interplay of both biotic and abiotic As redox cycling and biomethylation are occurring in most environments (Yin et al., 2022; Pongratz, 1998; Sharma and Sohn, 2009). These observations are in line with our results which demonstrate that As is found in both the oxidized and reduced forms in the microbial mat and the outflow channel water (Figure 3). One limitation of this analysis is that water and microbial mat samples underwent a nitric acid digestion prior to arsenic speciation and metal analysis via ICP-MS. It is possible that this digestion could have resulted in partial oxidation of As(III) to As(V) as well as some degradation of organic arsenicals. Although we were able to measure As(III), As(V), MMA, DMA, and arsenobetaine in the mat samples, future experiments would benefit from using alternative methods such as enzymatic digestion for liberation of metals from biologic material. Matrix matching the standard curves would also improve overall quantification of arsenical species.

Oxidation of As(III) is primarily utilized as a detoxification strategy for heterotrophic organisms but can also be utilized for energy metabolism by chemolithoautotrophic organisms (Zecchin et al., 2021). Microorganisms may also transform As to produce a range of organoarsenical species such as arsenosugars, arsenolipids, arsenobetaine, MMA, and DMA. These methylated organoarsenicals likely aid in the niche partitioning of complex environments (Chen and Rosen, 2020) and protect against osmotic stress and temperatures in extreme environments (Hoffmann et al., 2018). For example, arsenobetaine protects against high osmolarity and temperature in *Bacillus subtillis* (Hoffmann et al., 2018) and is likely to also play a cytoprotective role in the FS microbial organisms which experience high temperatures and osmotic stress (Table 1). MAGs identified the presence of *Roseiflexus* sp. *RS-1* in various layers and sites of FS which were found to be capable of organoarsenical efflux transport via ArsJ Supplementary Table S2. ArsJ is responsible for the efflux of arseno-3-phosphoglycerate (Shi et al., 2018), suggesting that additional organoarsenicals not measured within this analysis are present within the microbial mat system at FS. Future studies would benefit from a combined multi-omic approach that pairs genomic, metabolomic, and lipidomic analyses to provide a more global assessment of arsenic cycling and community interactions within the layers of the mat biofilm. Our data suggest that microorganisms within the mat maintain various and different arsenic resistance capabilities (Figure 7), indicating the presence of niche partition, reciprocal feeding and complex detoxification interactions between community members (Fritts et al., 2021; Berlanga et al., 2022; Prieto-Barajas et al., 2018). These data provide novel insights into potential interactive relationships between arsenic cycling and microbial community structure in a hot springs environment.

These findings support a model in which the microbial mat functions as a modular, stratified system shaped by environmental and geochemical gradients such as temperature, light availability, As and oxygen concentrations. These gradients likely govern microbial composition and interactions, while the microbial community also strongly influences these gradients as well. Although it is evident that the results presented here further our understanding of microbial mat biology and arsenic utilization by mat dwelling microorganisms, it is unclear how sampling time (both diurnal and seasonal) may affect these findings. Presumably, seasonal dynamics like discharge and temperature or spatial heterogeneity along the outflow channel or pool edges have the potential to alter this system, suggesting there is more spatial heterogeneity than that discussed within this manuscript. Additionally, microbial community profiles identified in this study are based on DNA, thus reflecting metabolic capabilities not necessarily metabolic activities. Future studies would benefit from confirming the genetic potential measure here with *in situ* activity and/or via multi-omic analysis combined with metabolic flux experiments. Overall, our conclusions align well with previous studies of phototrophic microbial mats in Yellowstone’s White Creek Drainage (Ward et al., 1998), reinforcing the role of physicochemical heterogeneity in shaping microbial ecology.

## Data Availability

The arsenic data presented in this study can be found at the following DOI: 10.6084/m9.figshare.31985211. The metagenomics data for this study have been deposited in the European Nucleotide Archive (ENA) at EMBL-EBI under accession number PRJEB111429 (https://www.ebi.ac.uk/ena/browser/view/PRJEB111429).

## References

[ref1] Anderson M. J. (2006). Distance-based tests for homogeneity of multivariate dispersions. Biometrics 62, 245–253. doi: 10.1111/j.1541-0420.2005.00440.x, 16542252

[ref2] Andres J. Bertin P. N. (2016). The microbial genomics of arsenic. FEMS Microbiol. Rev. 40, 299–322. doi: 10.1093/femsre/fuv050, 26790947

[ref3] Andrews S. FastQC: a Quality Control tool for high Throughput Sequence data (2010). Available online at: https://www.bioinformatics.babraham.ac.uk/projects/fastqc/

[ref4] Ascher J. Ceccherini M. T. Pantani O.-L. Agnelli A. Borgogni F. Guerri G. . (2009). Sequential extraction and genetic fingerprinting of a forest soil metagenome. Appl. Soil Ecol. 42, 176–181. doi: 10.1016/j.apsoil.2009.03.005

[ref6] Ball J. W. Nordstrom D. K. Jenne E. A. Vivit D. V. (1998). Chemical Analyses of hot springs, Pools, Geysers, and surface waters from Yellowstone National Park, Wyoming, and Vicinity, 1974–1975. Report. Reston, VA.

[ref7] Ball JW Nordstrom DK McCleskey RB Schoonen MAA Xu Y. Water-Chemistry and On-Site Sulfur-Speciation Data for Selected Springs in Yellowstone National Park, Wyoming, 1996–1998. Report. (2001). doi: 10.3133/ofr0149

[ref9] Bayles K. W. (2007). The biological role of death and lysis in biofilm development. Nat. Rev. Microbiol. 5, 721–726. doi: 10.1038/nrmicro1743, 17694072

[ref10] Berlanga M. Palau M. Guerrero R. (2022). Community homeostasis of coastal microbial mats from the Camargue during winter (cold) and summer (hot) seasons. Ecosphere 13:e3922. doi: 10.1002/ecs2.3922

[ref12] Bundschuh J. Maity J. P. (2015). Geothermal arsenic: Occurrence, Mobility and Environmental Implications, Renewable and Sustainable Energy Reviews. 42, 1214–1222. doi: 10.1016/j.rser.2014.10.092

[ref14] Cervantes C. ji G. Ramirez J. Silver S. (1994). Resistance to arsenic compounds in microorganisms. FEMS Microbiol. Rev. 15, 355–367. doi: 10.1111/j.1574-6976.1994.tb00145.x, 7848659

[ref15] Chen J. Hanke A. Tegetmeyer H. E. Kattelmann I. Sharma R. Hamann E. . (2017). Impacts of chemical gradients on microbial community structure. ISME J. 11, 920–931. doi: 10.1038/ismej.2016.175, 28094795 PMC5363838

[ref16] Chen J. Rosen B. P. (2020). The arsenic methylation cycle: how microbial communities adapted methylarsenicals for use as weapons in the continuing war for dominance. Front. Environ. Sci. 8. doi: 10.3389/fenvs.2020.00043

[ref17] Chung J. Y. Yu S. D. Hong Y. S. (2014). Environmental source of arsenic exposure. J. Prev. Med. Public Health 47, 253–257. doi: 10.3961/jpmph.14.036, 25284196 PMC4186553

[ref18] Colman D. R. Keller L. M. Arteaga-Pozo E. Andrade-Barahona E. St Clair B. Shoemaker A. . (2024). Covariation of hot spring geochemistry with microbial genomic diversity, function, and evolution. Nat. Commun. 15:7506. doi: 10.1038/s41467-024-51841-5, 39209850 PMC11362583

[ref19] Connon S. A. Koski A. K. Neal A. L. Wood S. A. Magnuson T. S. (2008). Ecophysiology and geochemistry of microbial arsenic oxidation within a high arsenic, circumneutral hot spring system of the Alvord Desert. FEMS Microbiol. Ecol. 64, 117–128. doi: 10.1111/j.1574-6941.2008.00456.x, 18318711

[ref20] Damer B. Deamer D. (2020). The hot spring hypothesis for an origin of life. Astrobiology 20, 429–452. doi: 10.1089/ast.2019.2045 31841362 PMC7133448

[ref21] Daoyan J. Zhiyong L. Zhihong L. (2022). Oxidation of as(III) by pressurized oxygen and the simultaneous precipitation of as(V) as scorodite in acidic sulfate solutions. Chem. Eng. J. 447:137395. doi: 10.1016/j.cej.2022.137395

[ref22] de Beer D. Stoodley P. Roe F. Lewandowski Z. (1994). Effects of biofilm structures on oxygen distribution and mass transport. Biotechnol. Bioeng. 43, 1131–1138. doi: 10.1002/bit.260431118, 18615526

[ref24] Drewniak L. Sklodowska A. (2013). Arsenic-transforming microorganisms and their role in biomining processes. Environ. Sci. Pollut. Res. 20, 7728–7739. doi: 10.1007/s11356-012-1449-0, 23299972 PMC3824281

[ref26] Felipe Benites L. Stephens T. G. Van Etten J. James T. Christian W. C. Barry K. . (2024). Hot springs viruses at Yellowstone National Park have ancient origins and are adapted to thermophilic hosts. Commun. Biol. 7:312. doi: 10.1038/s42003-024-05931-1, 38594478 PMC11003980

[ref27] Fournier R. O. (1989). Geochemistry and dynamics of the Yellowstone National Park hydrothermal system. Annu. Rev. Earth Planet. Sci. 17, 13–53. doi: 10.1146/annurev.ea.17.050189.000305

[ref28] Fritts R. K. McCully A. L. McKinlay J. B. (2021). Extracellular metabolism sets the table for microbial cross-feeding. Microbiol. Mol. Biol. Rev. 85. doi: 10.1128/mmbr.00135-20 PMC7849352 33441489

[ref29] Gurevich A. Saveliev V. Vyahhi N. Tesler G. (2013). QUAST: quality assessment tool for genome assemblies. Bioinformatics 29, 1072–1075. doi: 10.1093/bioinformatics/btt086, 23422339 PMC3624806

[ref30] Hoffmann T. Warmbold B. Smits S. H. J. Tschapek B. Ronzheimer S. Bashir A. . (2018). Arsenobetaine: an ecophysiologically important organoarsenical confers cytoprotection against osmotic stress and growth temperature extremes. Environ. Microbiol. 20, 305–323. doi: 10.1111/1462-2920.13999, 29159878

[ref31] Hurst C. J. (2022). Microbial Metabolism of Metals and Metalloids. Cham, Switzerland: Springer.

[ref11] Hyatt D. Chen G. L. Locascio P. F. Land M. L. Larimer F. W. Hauser L. J. (2010). Prodigal: prokaryotic gene recognition and translation initiation site identification. BMC bioinformatics, 11:119. doi: 10.1186/1471-2105-11-119 20211023 PMC2848648

[ref32] Jost L. (2006). Entropy and diversity. Oikos 113, 363–375. doi: 10.1111/j.2006.0030-1299.14714.x

[ref34] Kanehisa M. Sato Y. Morishima K. (2016). BlastKOALA and GhostKOALA: KEGG tools for functional characterization of genome and metagenome sequences. J. Mol. Biol. 428, 726–731. doi: 10.1016/j.jmb.2015.11.006, 26585406

[ref35] Karn S. K. Pan X. (2016). Role of *Acinetobacter* sp. in arsenite as (III) oxidation and reducing its mobility in soil. Chem. Ecol. 32, 460–471. doi: 10.1080/02757540.2016.1157174

[ref36] Keren R. Méheust R. Santini J. M. Thomas A. West-Roberts J. Banfield J. F. . (2022). Global genomic analysis of microbial biotransformation of arsenic highlights the importance of arsenic methylation in environmental and human microbiomes. Comput. Struct. Biotechnol. J. 20, 559–572. doi: 10.1016/j.csbj.2021.12.040, 36284711 PMC9582695

[ref37] Kers J. G. Saccenti E. (2021). The power of microbiome studies: some considerations on which alpha and beta metrics to use and how to report results. Front. Microbiol. 12:796025. doi: 10.3389/fmicb.2021.796025 35310396 PMC8928147

[ref38] Kostešić E. Mitrović M. Kajan K. Marković T. Hausmann B. Orlić S. . (2023). Microbial diversity and activity of biofilms from Geothermal Springs in Croatia. Microb. Ecol. 86, 2305–2319. doi: 10.1007/s00248-023-02239-1, 37209180 PMC10640420

[ref39] Kozubal M. A. Macur R. E. Jay Z. J. Beam J. P. Malfatti S. A. Tringe S. G. . (2012). Microbial iron cycling in acidic geothermal springs of Yellowstone National Park: integrating molecular surveys, geochemical processes, and isolation of novel fe-active microorganisms. Front. Microbiol. 3:109. doi: 10.3389/fmicb.2012.00109, 22470372 PMC3312321

[ref40] Landrum J. T. Bennett P. C. Engel A. S. Alsina M. A. Pastén P. A. Milliken K. (2009). Partitioning geochemistry of arsenic and antimony, El Tatio geyser field, Chile. Appl. Geochem. 24, 664–676. doi: 10.1016/j.apgeochem.2008.12.024

[ref41] Langner H. W. Jackson C. R. McDermott T. R. Inskeep W. P. (2001). Rapid oxidation of Arsenite in a hot spring ecosystem, Yellowstone National Park. Environ. Sci. Technol. 35, 3302–3309. doi: 10.1021/es0105562, 11529568

[ref42] Larson J. Tokmina-Lukaszewska M. Fausset H. Spurzem S. Cox S. Cooper G. . (2023). Arsenic exposure causes global changes in the metalloproteome of *Escherichia coli*. Microorganisms 11, 1–15. doi: 10.3390/microorganisms11020382, 36838347 PMC9965246

[ref43] Leal-Acosta M. L. Shumilin E. Mirlean N. Sapozhnikov D. Gordeev V. (2010). Arsenic and Mercury Contamination of Sediments of Geothermal Springs, Mangrove Lagoon and the Santispac Bight, Bahía Concepción, Baja California Peninsula, Bull. Environ. Contam. Toxicol. 85, 609–613. doi: 10.1007/s00128-010-0135-5 21107528

[ref44] Legendre P. Gallagher E. D. (2001). Ecologically meaningful transformations for ordination of species data. Oecologia 129, 271–280. doi: 10.1007/s004420100716, 28547606

[ref46] Li H. (2018). Minimap2: pairwise alignment for nucleotide sequences. Bioinformatics 34, 3094–3100. doi: 10.1093/bioinformatics/bty191, 29750242 PMC6137996

[ref47] Li D. Luo R. Liu C. M. Leung C. M. Ting H. F. Sadakane K. . (2016). MEGAHIT v1.0: a fast and scalable metagenome assembler driven by advanced methodologies and community practices. Methods 102, 3–11. doi: 10.1016/j.ymeth.2016.02.020, 27012178

[ref48] Love M. I. Huber W. Anders S. (2014). Moderated estimation of fold change and dispersion for RNA-seq data with DESeq2. Genome Biol. 15:550. doi: 10.1186/s13059-014-0550-8, 25516281 PMC4302049

[ref50] Maity J. P. Liu C.-C. Nath B. Bundschuh J. Kar S. Jean J.-S. . (2011). Biogeochemical Characteristics of Kuan-Tzu-Ling, Chung-Lun and Bao-Lai hot springs in southern Taiwan, J Environ Sci Health A Tox Hazard Subst Environ Eng. 46, 1207–1217. doi: 10.1080/10934529.2011.598788 21879853

[ref51] Malard L. A. Guisan A. (2023). Into the microbial niche. Trends Ecol. Evol. 38, 936–945. doi: 10.1016/j.tree.2023.04.015, 37236880

[ref52] Malina T. Koehorst R. Bína D. Pšenčík J. van Amerongen H. (2021). Superradiance of bacteriochlorophyll c aggregates in chlorosomes of green photosynthetic bacteria. Sci. Rep. 11:8354. doi: 10.1038/s41598-021-87664-3, 33863954 PMC8052352

[ref54] Messens J. Silver S. (2006). Arsenate reduction: thiol Cascade chemistry with convergent evolution. J. Mol. Biol. 362, 1–17. doi: 10.1016/j.jmb.2006.07.002, 16905151

[ref55] Moreira C. M. Duarte F. A. Lebherz J. Pozebon D. Flores E. M. M. Dressler V. L. (2011). Arsenic speciation in white wine by LC–ICP–MS. Food Chem. 126, 1406–1411. doi: 10.1016/j.foodchem.2010.11.120

[ref58] Nguyen M. H. Pham T. D. Nguyen T. L. Vu H. A. Ta T. T. Tu M. B. . (2018). Speciation analysis of arsenic compounds by HPLC-ICP-MS: application for human serum and urine. J. Anal. Methods Chem. 2018:9462019. doi: 10.1155/2018/9462019, 30538885 PMC6258103

[ref59] Oksanen J Simpson G Blanchet FG Kindt R Legendre P Minchin P vegan Community Ecology Package Version 2.6–2 April 2022 (2022)

[ref60] Ordóñez E. Van Belle K. Roos G. De Galan S. Letek M. Gil J. A. . (2009). Arsenate reductase, mycothiol, and mycoredoxin concert thiol/disulfide exchange. J. Biol. Chem. 284:19286650, 15107–15116. doi: 10.1074/jbc.M900877200 PMC2685692 19286650

[ref61] Ospino M. C. Kojima H. Fukui M. (2019). Arsenite oxidation by a newly isolated Betaproteobacterium possessing arx genes and diversity of the arx gene cluster in bacterial genomes. Front. Microbiol. 10, 1–16. doi: 10.3389/fmicb.2019.01210 31191509 PMC6549141

[ref62] Pande V. Pandey S. C. Sati D. Bhatt P. Samant M. (2022). Microbial interventions in bioremediation of heavy metal contaminants in agroecosystem. Front. Microbiol. 13. doi: 10.3389/fmicb.2022.824084 PMC9120775 35602036

[ref63] Parks D. H. Imelfort M. Skennerton C. T. Hugenholtz P. Tyson G. W. (2015). CheckM: assessing the quality of microbial genomes recovered from isolates, single cells, and metagenomes. Genome Res. 25, 1043–1055. doi: 10.1101/gr.186072.114, 25977477 PMC4484387

[ref64] Peach J. T. Mueller R. C. Skorupa D. J. Mesle M. M. Kanta S. Boltinghouse E. . (2022). Longitudinal analysis of the five sisters hot springs in Yellowstone National Park reveals a dynamic thermoalkaline environment. Nature Research. 12, 1–15. doi: 10.1038/s41598-022-22047-w PMC9636164 36333441

[ref65] Peipoch M. Miller S. R. Antao T. R. Valett H. M. (2019). Niche partitioning of microbial communities in riverine floodplains. Sci. Rep. 9:16384. doi: 10.1038/s41598-019-52865-4, 31705005 PMC6841707

[ref66] Perillo V. L. Nute M. Sapoval N. Curry K. D. Golia L. Yin Y. . (2025). A survey of computational approaches for characterizing microbial interactions in microbial mats. Genome Biol. 26:168. doi: 10.1186/s13059-025-03634-2, 40524188 PMC12168310

[ref67] Podar P. T. Yang Z. Björnsdóttir S. H. Podar M. (2020). Comparative analysis of microbial diversity across temperature gradients in hot springs from Yellowstone and Iceland. Front. Microbiol. 11, 1–16. doi: 10.3389/fmicb.2022.824084 32760379 PMC7372906

[ref68] Pongratz R. (1998). Arsenic speciation in environmental samples of contaminated soil. Sci. Total Environ. 224, 133–141. doi: 10.1016/s0048-9697(98)00321-0

[ref70] Prieto-Barajas C. M. Valencia-Cantero E. Santoyo G. (2018). Microbial mat Ecosystems: Structure types, Functional Diversity, and Biotechnological Application, Electron. J. Biotechnol. 31, 48–56. doi: 10.1016/j.ejbt.2017.11.001

[ref71] Roswell M. Dushoff J. Winfree R. (2021). A conceptual guide to measuring species diversity. Oikos 130, 321–338. doi: 10.1111/oik.07202

[ref72] Roy C. Rameez M. J. Haldar P. K. Peketi A. Mondal N. Bakshi U. . (2020). Microbiome and ecology of a hot spring-microbialite system on the trans-Himalayan plateau. Sci. Rep. 10:5917. doi: 10.1038/s41598-020-62797-z, 32246033 PMC7125080

[ref73] Russell M. J. (1996). The Generation at hot springs of Sedimentary ore Deposits, Microbialites and life, 199–214. Amsterdam, Netherlands: Elsevier Science BV.

[ref74] Saidi-Mehrabad A. Neuberger P. Cavaco M. Froese D. Lanoil B. (2020). Optimization of subsampling, decontamination, and DNA extraction of difficult peat and silt permafrost samples. Sci. Rep. 10:14295. doi: 10.1038/s41598-020-71234-0, 32868827 PMC7459103

[ref75] Santini J. M. Sly L. I. Schnagl R. D. Macy J. M. (2000). A new chemolithoautotrophic arsenite-oxidizing bacterium isolated from a gold mine: phylogenetic, physiological, and preliminary biochemical studies. Appl. Environ. Microbiol. 66, 92–97. doi: 10.1128/AEM.66.1.92-97.2000, 10618208 PMC91790

[ref76] Sharma V. K. Sohn M. (2009). Aquatic arsenic: toxicity, speciation, transformations, and remediation. Environ. Int. 35, 743–759. doi: 10.1016/j.envint.2009.01.005, 19232730

[ref77] Shi L.-D. Guo T. Lv P.-L. Niu Z.-F. Zhou Y.-J. Tang X.-J. . (2020). Coupled anaerobic methane oxidation and reductive arsenic mobilization in wetland soils. Nat. Geosci. 13, 799–805. doi: 10.1038/s41561-020-00659-z

[ref78] Shi K. Li C. Rensing C. Dai X. Fan X. Wang G. (2018). Efflux transporter ArsK is responsible for bacterial resistance to arsenite, antimonite, trivalent roxarsone, and methylarsenite. Appl. Environ. Microbiol. 84:e01842-18. doi: 10.1128/AEM.01842-18, 30315082 PMC6275340

[ref79] Song X. Li Y. Stirling E. Zhao K. Wang B. Zhu Y. . (2022). AsgeneDB: a curated orthology arsenic metabolism gene database and computational tool for metagenome annotation. NAR Genom. Bioinform. 4:lqac080. doi: 10.1093/nargab/lqac080, 36330044 PMC9623898

[ref80] Stauffer RE Jenne EA Ball JW. Chemical Studies of Selected trace Elements in hot-spring Drainages of Yellowstone National Park. Report. (1980).

[ref81] Stolz J. F. Oremland R. S. (1999). Bacterial respiration of arsenic and selenium. FEMS Microbiol. Rev. 23, 615–627. doi: 10.1111/j.1574-6976.1999.tb00416.x, 10525169

[ref82] Suarez C. Piculell M. Modin O. Langenheder S. Persson F. Hermansson M. (2019). Thickness determines microbial community structure and function in nitrifying biofilms via deterministic assembly. Sci. Rep. 9:5110. doi: 10.1038/s41598-019-41542-1, 30911066 PMC6434030

[ref83] Tamaki S. Frankenberger W. T. Jr. (1992). Environmental biochemistry of arsenic. Rev. Environ. Contam. Toxicol. 124, 79–110. 1732996 10.1007/978-1-4612-2864-6_4

[ref84] Tanmoy P. Chakraborty A. Islam E. Mukherjee S. K. (2018). Arsenic bioremediation potential of arsenite-oxidizing *Micrococcus* sp. KUMAs15 isolated from contaminated soil. Pedosphere 28, 299–310. doi: 10.1016/S1002-0160(17)60493-4

[ref85] Tian B. Hua Y. (2010). Carotenoid biosynthesis in extremophilic Deinococcus–Thermus bacteria. Trends Microbiol. 18, 512–520. doi: 10.1016/j.tim.2010.07.007, 20832321

[ref86] Velásquez L. Dussan J. (2009). Biosorption and bioaccumulation of heavy metals on dead and living biomass of *Bacillus sphaericus*. J. Hazard. Mater. 167, 713–716. doi: 10.1016/j.jhazmat.2009.01.044, 19201532

[ref87] Visscher P. T. Gallagher K. L. Bouton A. Farias M. E. Kurth D. Sancho-Tomás M. . (2020). Modern arsenotrophic microbial mats provide an analogue for life in the anoxic Archean. Commun. Earth Environ. 1:24. doi: 10.1038/s43247-020-00025-2

[ref88] Wang Y. Li P. Guo Q. Jiang Z. Liu M. (2018). Environmental biogeochemistry of high arsenic geothermal fluids. Appl. Geochem. 97, 81–92. doi: 10.1016/j.apgeochem.2018.07.015

[ref89] Ward D. M. Ferris M. J. Nold S. C. Bateson M. M. (1998). A Natural View of Microbial Biodiversity within Hot Spring Cyanobacterial Mat Communities, 1353–1370. 10.1128/mmbr.62.4.1353-1370.1998 PMC98949 9841675

[ref90] Wilkie J. A. Hering J. G. (1998). Rapid oxidation of geothermal arsenic(III) in streamwaters of the eastern Sierra Nevada. Environ. Sci. Technol. 32, 657–662. doi: 10.1021/es970637r

[ref91] Wood JM Urbaniak C Parker C Singh NK Wong S Arumugam A. (2024). Assessing Microbial Diversity in Yellowstone National Park hot springs using a field Deployable Automated Nucleic acid Extraction system. Front. Ecol. Evol. 12:1306008. doi: 10.3389/fevo.2024.1306008

[ref92] Wörmer L. Gajendra N. Schubotz F. Matys E. D. Evans T. W. Summons R. E. . (2020). A Micrometer-scale Snapshot on Phototroph Spatial Distributions: mass Spectrometry Imaging of Microbial mats in Octopus Spring, Yellowstone National Park, Geobiology. 18, 742–759. doi: 10.1111/gbi.12411 32936514

[ref93] Yin S. Zhang X. Yin H. Zhang X. (2022). Current knowledge on molecular mechanisms of microorganism-mediated bioremediation for arsenic contamination: a review. Microbiol. Res. 258:126990. doi: 10.1016/j.micres.2022.126990, 35190347

[ref94] Zecchin S. Crognale S. Zaccheo P. Fazi S. Amalfitano S. Casentini B. . (2021). Adaptation of Microbial Communities to Environmental Arsenic and Selection of Arsenite-Oxidizing Bacteria From Contaminated Groundwaters. Front. Microbiol. 12:634025. doi: 10.3389/fmicb.2021.634025 33815317 PMC8017173

[ref95] Zhu Y.-G. Xue X.-M. Kappler A. Rosen B. P. Meharg A. A. (2017). Linking genes to microbial biogeochemical cycling: lessons from arsenic. Environ. Sci. Technol. 51, 7326–7339. doi: 10.1021/acs.est.7b00689, 28602082 PMC5871744

