# Supplementary material for: Arsenic detoxification within thermo-alkaline biofilms
Source: Front Microbiol. 2026 Apr 30;17:1783099. doi: 10.3389/fmicb.2026.1783099 (PMC13171826; doi:10.3389/fmicb.2026.1783099)
Supplement: Supplementary file 1 [file Data_Sheet_1.DOCX]

**Supplementary Material**

Arsenic detoxification within thermo-alkaline biofilms

Gwendolyn Cooper^a,b,†^, Stephanie H. Ayotte^a,c,d, †^, Martina L. Du^a,c,e^, Jessica D. Wood^a,c,f^, Breuklyn Opp^a,c,e^, Brian Bothner^b^, Brent M. Peyton^a,c,e,*^

^a^ Thermal Biology Institute, Montana State University, Bozeman, MT USA 59717

^b^ Department of Chemistry and Biochemistry, Montana State University, Bozeman, MT USA 59717

^c^ Center for Biofilm Engineering, Montana State University, Bozeman, MT USA 59717

^d^ Department of Civil Engineering, Montana State University, Bozeman, MT USA59717

^e^ Department of Chemical and Biological Engineering, Montana State University, Bozeman, MT USA 59717

^f^ Department of Microbiology and Cell Biology, Montana State University, Bozeman, MT USA59717

^*^ Corresponding author [bpeyton@montana.edu](mailto:bpeyton@montana.edu)

^†^ These authors contributed equally to this work.


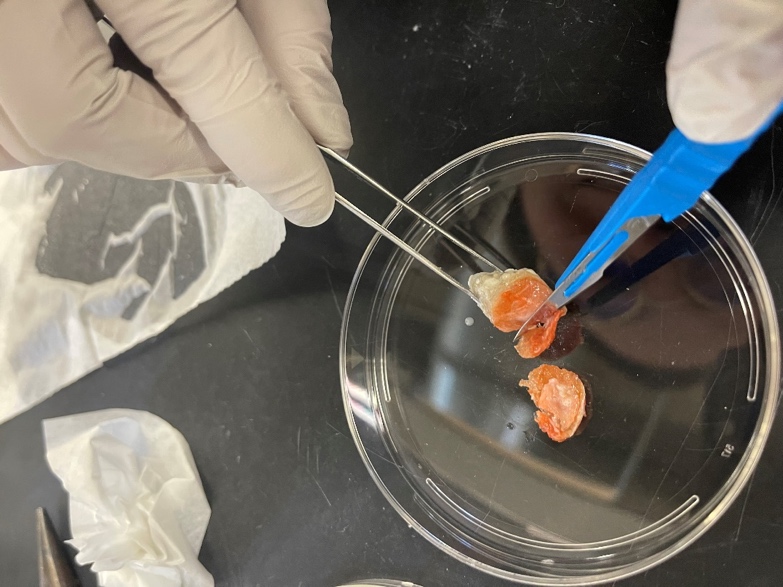

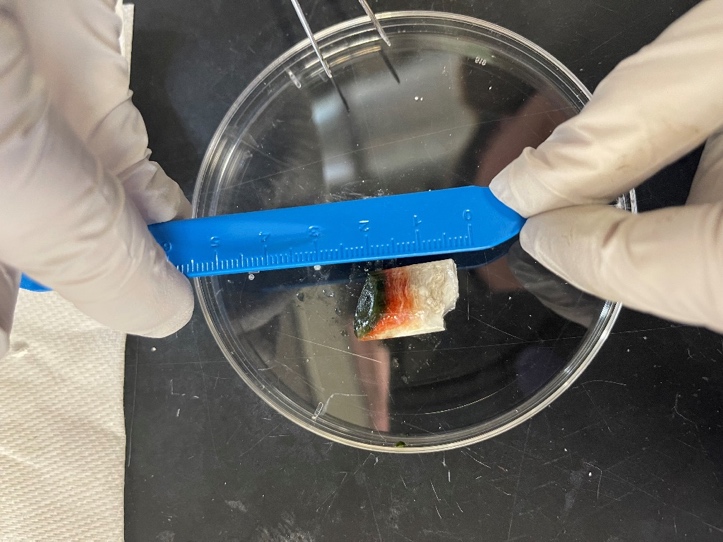


Fig. S1 Sectioning of mat biofilm for DNA extraction.

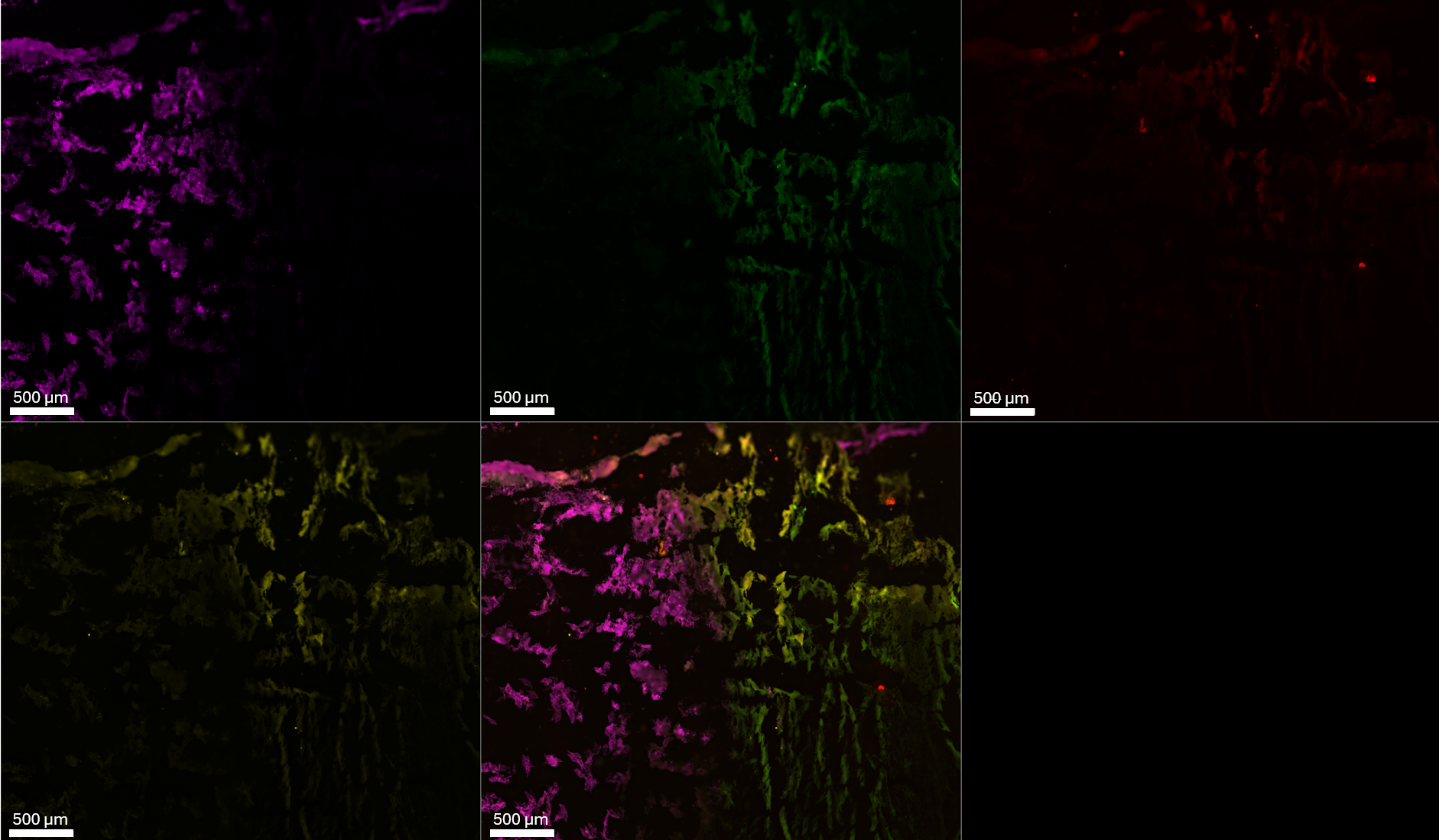


Figure S2. Zoomed in sections of the mat from figure 7b. The intermixed fluorescence in multiple regions in the microbial mat suggests close physical proximity and potential metabolic cooperation or competition between microbial groups. Fluorescence signals shown were captured using Cy5, GFP, RFP, YFP filters, reflecting natural autofluorescence and pigment emissions. The last image is an overlay of all fluorescent channels.
